# Enhanced Effects of Intermittent Fasting by Magnetic Fields in Severe Diabetes

**DOI:** 10.34133/research.0468

**Published:** 2024-09-05

**Authors:** Ying Wang, Chuanlin Feng, Biao Yu, Junjun Wang, Weili Chen, Chao Song, Xinmiao Ji, Ruowen Guo, Guofeng Cheng, Hanxiao Chen, Xinyu Wang, Lei Zhang, Zhiyuan Li, Jialiang Jiang, Can Xie, Haifeng Du, Xin Zhang

**Affiliations:** ^1^ High Magnetic Field Laboratory, CAS Key Laboratory of High Magnetic Field and Ion Beam Physical Biology, Hefei Institutes of Physical Science, Chinese Academy of Sciences, Hefei, Anhui, China.; ^2^ Science Island Branch of Graduate School, University of Science and Technology of China, Hefei, Anhui, China.; ^3^ NHC Key Laboratory of Study on Abnormal Gametes and Reproductive Tract, Anhui Medical University, Hefei, Anhui, China.; ^4^ Institutes of Physical Science and Information Technology, Anhui University, Hefei, Anhui, China.; ^5^ Medical Research Council (MRC) Protein Phosphorylation and Ubiquitylation Unit, School of Life Sciences, University of Dundee, Dundee, UK.

## Abstract

Intermittent fasting (IF) is a convenient dietary intervention for multiple diseases, including type 2 diabetes. However, whether it can be used as a long-term antidiabetic approach is still unknown. Here, we confirm that IF alone is beneficial for both moderate and severe diabetic mice, but its antidiabetic effects clearly diminish at later stages, especially for severe diabetic db/db mice, which have obviously impaired autophagy. We found that static magnetic fields can directly promote actin assembly and boost IF-induced autophagy. Consequently, the pancreatic islet and liver were improved, and the antidiabetic effects of IF were boosted. In fact, at later stages, combined static magnetic field and IF could reduce the blood glucose level of moderate type 2 diabetic mice by 40.5% (*P* < 0.001) and severe type 2 diabetes by 34.4% (*P* < 0.05), when IF alone no longer has significant blood glucose reduction effects. Therefore, although IF is generally beneficial for diabetes, our data reveal its insufficiency for late-stage diabetes, which can be compensated by a simple, noninvasive, long-lasting, and nonpharmacological strategy for effective long-term diabetic control.

## Introduction

Diabetes is a metabolic disorder characterized by high blood glucose levels, which is becoming a global epidemic, affecting over 500 million people worldwide [1]. Type 2 diabetes stands as the most prevalent form, accounting for over 90% of all diabetes cases globally. Type 2 diabetes is typically associated with insulin secretion defects, insulin resistance, and the death of pancreatic β cells [2,3]. Therefore, it is essential to develop strategies that can improve diabetes by mitigating impaired pancreatic β cell function and metabolic disturbances, especially for long-term management of diabetes.

Numerous investigations have documented the improvement of type 2 diabetes through dietary modifications, including intermittent fasting (IF), which has gained attention as an innovative and promising dietary intervention. Compared to other fasting plans, IF offers a simpler and more convenient approach for implementation and maintenance. Extensive research has revealed various benefits associated with IF, such as extended lifespan [4], improved cardiovascular health [5], and delayed progression of neurodegenerative diseases [6]. In particular, it has demonstrated significant effects on obesity and diabetes, including weight reduction [7], improved insulin sensitivity and lowered blood glucose levels [8], alleviated diabetes-related cognitive impairments [9], and retinopathy [10]. In addition, IF can help patients with diabetes reduce their medication dosages [11], making it a promising diabetes managing strategy that can be used in combination with other methods. However, the long-term effect of IF is rarely studied. In terms of animal experiments, Patel et al. [12] conducted an interesting study. They found that IF for 16 weeks in 2 polygenic diabetic mice with different severities can restore β cell function and significantly improve hyperglycemia and insulin sensitivity. However, some clinical studies indicate that IF may not have satisfactory improvement effects on blood glucose levels, insulin sensitivity, or insulin resistance in obese individuals. For example, Bhutani et al. [13] found that 12 weeks of alternate day fasting treatment had no significant effect on fasting glucose, fasting insulin, and homeostasis model assessment of insulin resistance (HOMA-IR) in obese adults. The report by Trepanowski et al. [14] showed that alternate day fasting treatment for 12 months did not show better effects on indicators such as blood glucose and insulin resistance in obese individuals compared to 6 months.

Interestingly, in recent years, electromagnetic fields have been shown to be a potential physical tool for modulating diabetes. For example, Huang et al. [15] discovered that a specially designed current-generating device producing direct current can stimulate insulin secretion and improve hyperglycemic symptoms in type 1 diabetes. Carter et al. [16] demonstrated that a 3-mT static magnetic field (SMF) combined with an electric field could improve insulin resistance in high-fat diet (HFD)-induced type 2 diabetes mice and genetic mutant db/db mice by regulating the redox system. Our group also found that a 0.1-T SMF could alleviate diabetes in HFD + streptozotocin (STZ)-induced type 2 diabetes by modulating iron metabolism and gut microbiota [17]. Furthermore, Lv et al. [18] recently discovered that an 8-week exposure to a 0.4- to 0.7-T SMF significantly improved liver function in diabetic mice. SMFs have also shown promising results in ameliorating diabetic complications [19,20]. Therefore, magnetic fields have a potential to be served as a noninvasive and safe physical method for diabetes.

Since IF has been shown to be able to help patients with diabetes reduce their medication dosages [11], it shows great potential as a combinational strategy. In this study, to investigate the long-term antidiabetic effects of IF, as well as the potential effects of IF combined with SMF, we chose 2 different mouse models of different blood glucose levels, representing moderate versus severe type 2 diabetes. Our results demonstrate that although IF alone works effectively to reduce blood glucose level in early-stage diabetes, it stops working on late-stage severe diabetes, which seems to be related to insufficient autophagy at later stage. Using SMF as a noninvasive physical tool to promote actin cytoskeleton assembly, we are able to boost IF-induced autophagy and restore pancreatic islet and liver function, which significantly increases the antidiabetic efficacy of IF and leads to satisfactory antidiabetic effects on both moderate and severe type 2 diabetes mice.

## Results

### SMF promotes the blood glucose reduction effects of IF in moderate diabetic mice

**Fig. 1.**
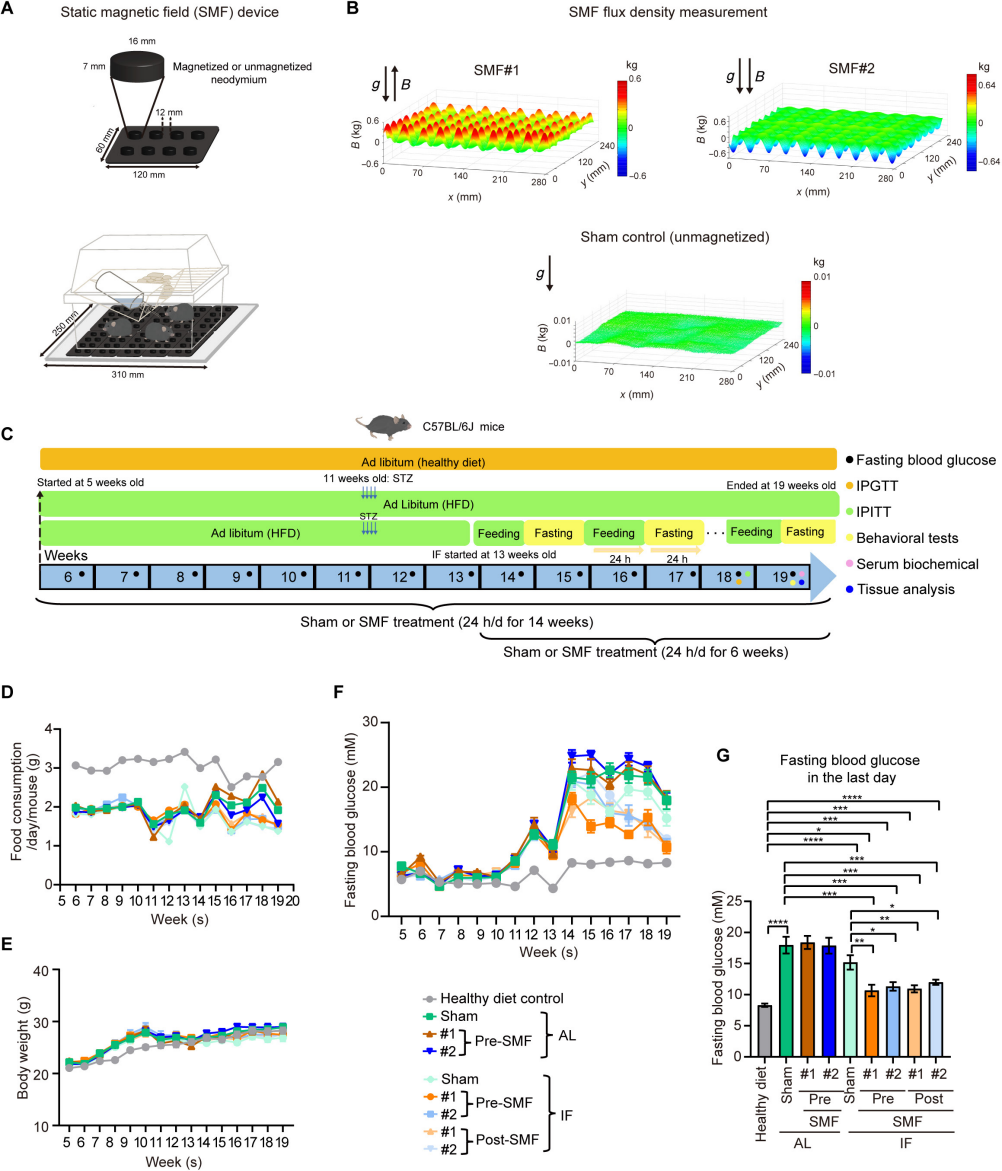
SMFs promote the blood glucose reduction effects of IF in moderate diabetic mice. (A) The SMF devices. (B) Magnetic fields distribution in the mouse exposure area, 8 mm above the magnetized (SMF#1 and SMF#2) or unmagnetized (sham control) plates. (C) Experimental design. HFD + STZ-induced C57BL/6J diabetic mice were used. (D) Food consumption of mice (*n* = 8 to 10 mice per group). (E) Body weight of mice (*n* = 8 to 10 mice per group). (F) Fasting blood glucose curves of mice [*n* = 8 to 10 mice per group, for (D) to (F) weeks indicating the mouse age]. Fasting time, 6 h. (G) Fasting blood glucose of mice on the last day of the experiment (*n* = 8 to 10 mice per group). All data are presented as means ± SEM and analyzed by GraphPad Prism 9.0. *P < 0.05, **P < 0.01, ***P < 0.001, and ****P < 0.0001, 2-tailed Student’s *t* test (G).

To investigate the single or combined effects of IF and SMF on diabetes, we used magnetic field devices made of cylindrical permanent magnets (Fig. 1A). Identical setting with unmagnetized NdFeB was used as sham control. The cylindrical permanent magnets are arranged with the north pole facing up (SMF#1) or south pole facing up (SMF#2). We used a magnetic analyzer to scan the field distribution horizontally at 8 mm above the magnetic plate (Fig. 1B), approximately where the mouse abdomen is when they are in the cage, and the maximum magnetic flux density here is about 20 to 60 mT.

We first used HFD + STZ to induce diabetes in mice, which is a commonly used method to simulate the natural process and metabolic characteristics of human diabetes [21–23]. The C57BL/6J mice were fed with healthy diet or HFD with ad libitum (AL) or IF pattern. STZ was injected at 11th week to induce moderate diabetes since it can increase the mouse 6-h fasting blood glucose levels to ~20 mM, which will be much lower than the severe diabetic db/db mice. IF of HFD was initiated at 13th week. We set up 2 different SMF treatment procedures to examine the potential “preventive” and “therapeutic” effects of SMFs. One group of mice, labeled as “pre-SMF”, started SMF treatment at 5 weeks of age to test potential preventive effects”, and the total treatment time was 14 weeks. Another group, labeled “post-SMF”, started SMF treatment after diabetes symptoms had developed, when the mice were 13 weeks old, to test potential treatment effects, and the total treatment time was 6 weeks (Fig. 1C). Our results show that the food or water consumption was not affected by SMF treatment (Fig. 1D and Fig. S1A) nor the mouse body weight (Fig. 1E and Fig. S1B). Consistent with previous reports [24,25], STZ injection caused weight loss in mice, so the mice in the HFD + STZ group continued to gain weight during the early feeding period of HFD but lost weight after injection of STZ and ultimately had similar weight as the control group mice (Fig. 1E).

To monitor the diabetic progression and the effects of IF and/or SMFs, we measured their fasting blood glucose levels every week. In contrast, SMF combined with IF significantly reduced the fasting blood glucose levels in mice (Fig. 1F and Fig. S1C). Both the “pre” and “post” groups of SMF with IF showed favorable effects (Fig. S1C). On the last day of the experiment, the fasting blood glucose levels were ~8 mM in healthy diet control, ~18 mM in the AL group, ~15 mM in the IF alone group, and the SMF + IF group decreased to~10 mM (Fig. 1G).

### SMF promotes IF effects on pancreatic islet and fatty liver in moderate diabetic mice

**Fig. 2.**
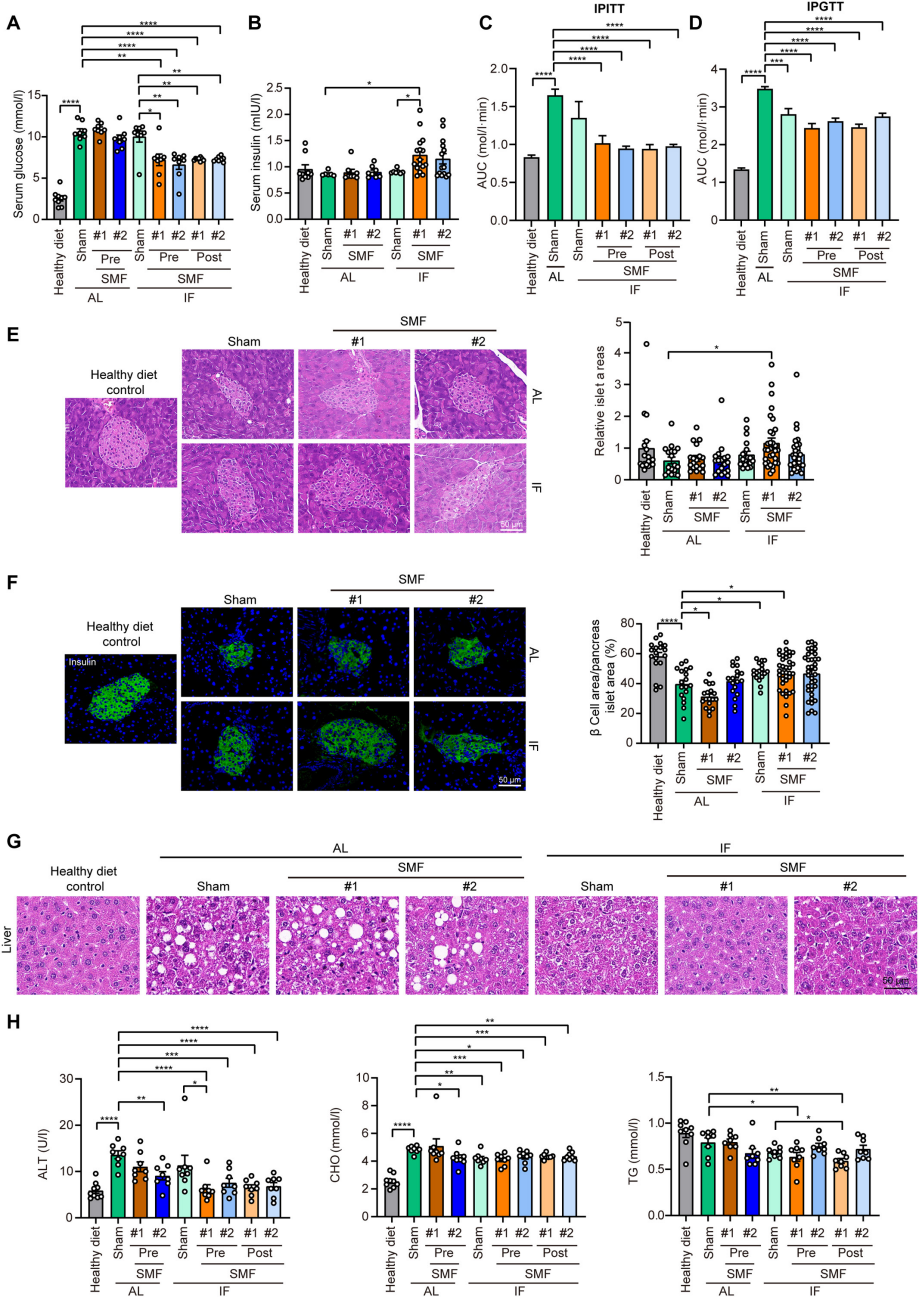
SMFs promote improvement effects of IF on moderate diabetic mouse pancreatic islet and liver function. HFD +STZ-induced C57BL/6J diabetic mice were used. (A) Serum glucose level of mice (*n* = 8 to 9 mice per group) after fasting for ~12 h. (B) Serum insulin level of mice (*n* = 6 to 16 mice per group). (C) Blood glucose AUC of IPITTs (*n* = 8 to 9 mice per group). (D) AUC of IPGTTs (*n* = 8 to 9 mice per group). (E) Representative H&E images of mouse pancreatic islets and quantification of pancreatic islet area (*n* = 18 to 36 islets per group and 3 mice per group). (F) Representative immunofluorescence images of insulin in mouse pancreatic islets (green, anti-insulin) and quantification of beta cell area (*n* = 18 to 36 islets per group and 3 mice per group). (G) Representative H&E images of mouse liver (*n* = 3 mice per group). (H) Serum alanine aminotransferase (ALT), cholesterol (CHO), and triglyceride (TG) of mice (*n* = 8 to 9 mice per group). All data are presented as means ± SEM and analyzed by GraphPad Prism 9.0. *P < 0.05, **P < 0.01, ***P < 0.001, and ****P < 0.0001, 2-tailed Student’s *t* test (A to F and H).

Other than elevated fasting blood glucose level, there are multiple other diabetic symptoms, such as impaired pancreatic islet and fatty liver. We first examined the mouse serum glucose levels (Fig. 2A) and insulin levels (Fig. 2B) and found that IF alone no longer had improvement effects on these 2 indicators after 6 weeks of treatment. However, the insulin tolerance test (ITT) (Fig. 2C and Fig. S1D and E) and glucose tolerance test (GTT) (Fig. 2D and Fig. S1F and G) show that IF alone did improve the mouse insulin tolerance, but these effects can be promoted by SMF, no matter pre- or post-SMF treatment (Fig. 2C and D). Tissue examination confirms that the pancreatic islet areas were restored in SMF + IF group (Fig. 2E) and the pancreatic insulin levels were also restored by IF and SMF + IF (Fig. 2F).

Furthermore, type 2 diabetes is often associated with fatty liver and various complications. Our hematoxylin–eosin (H&E) staining results show that the livers of HFD + STZ-induced diabetic mice exhibited a significant amount of vacuolation and lipid accumulation, while IF can significantly reduce hepatic lipid accumulation and improve liver function (Fig. 2G and H and Fig. S2). Moreover, IF + SMF can further improve the liver function and ameliorate HFD + STZ-induced abnormal liver functions and blood lipid levels (Fig. 2G and H), and there is a trend toward promoting exploratory behavior and activity abilities (Fig. S3) in HFD + STZ-induced C57BL/6J diabetic mice.

### SMF + IF prevents hyperglycemia development in severe diabetic mice and prolongs their survival time

**Fig. 3.**
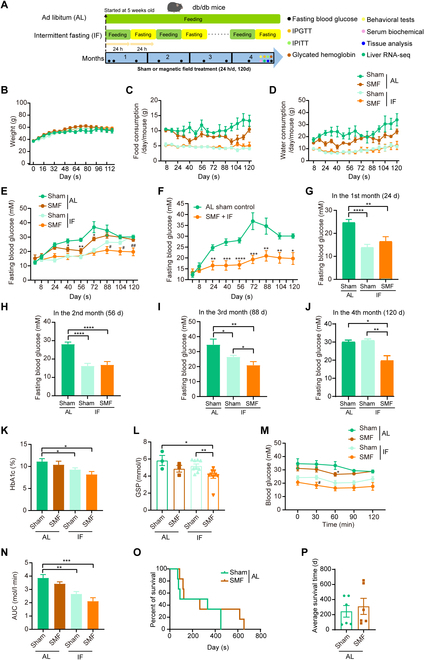
SMF combined with IF improves hyperglycemia and survival in severe db/db diabetic mice. Genetically diabetic BKS-Leprdb/J (db/db) mice were used. (A) Experimental design. (B) Body weight curves (*n* = 6 to 9 mice per group). (C) Food consumption curves (*n* = 6 to 9 mice per group). (D) Water consumption curves (*n* = 6 to 9 mice per group). (E) Fasting blood glucose level curves of all 4 groups (*n* = 6 to 9 mice per group). (F) Fasting blood glucose level curves of AL sham control versus SMF + IF groups (*n* = 6 to 9 mice per group). (G) Fasting blood glucose level in the first month of experiment (*n* = 9 mice per group). (H) Fasting blood glucose level in the second month of experiment (*n* = 9 mice per group). (I) Fasting blood glucose level in the third month of experiment (*n* = 7 to 9 mice per group). (J) Fasting blood glucose level in the fourth month of experiment (*n* = 6 to 9 mice per group). (K) HbA1c (*n* = 6 to 9 mice per group). (L) GSP (*n* = 3 to 9 mice per group). (M) IPITT (*n* = 6 to 9 mice per group). (N) Blood glucose AUC of IPITT (*n* = 6 to 9 mice per group). (O) Survival curves of AL mice with or without SMF treatment (*n* = 6 mice per group). (P) Average survival time of AL mice with or without SMF treatment (*n* = 6 mice per group). All data are presented as means ± SEM and analyzed by GraphPad Prism 9.0. Asterisk represents the significance between AL sham control and SMF + AL, and number symbol represents the significance between IF sham control and SMF + IF. *P < 0.05, **P < 0.01, ***P < 0.001, ****P < 0.0001, #*P* < 0.05, and ##*P* < 0.01, 2-tailed Student’s *t* test (E to N).

Since the above results show that IF alone can benefit moderate type 2 diabetes mice at early stages but the effect decreased at later stages, we next used the db/db mice, a genetically diabetic mouse model, to monitor the whole development process of diabetes. The db/db mice were subjected to IF or normal AL diet from the very beginning of the experiment, when the mice were 5 weeks old, along with either sham or SMF treatment (Fig. 3A). Since the 2 different types of magnetic settings (SMF#1 and SMF#2) and pre- or post-SMF treatment did not show obvious differences in previous moderate diabetic mouse experiments, here, we only examined SMF#1 in the db/db mice and labeled as SMF from now on. The processing time of SMF is consistent with IF, with a total of 120 d, to explore the effect of SMF + IF on the development of diabetes.

For both types of mouse models, the IF, SMF, or their combination did not affect the body weight (Figs. 1E and 3B), which is probably due to the young age of mice (5 weeks old) we used. However, the excessive food (Fig. 3C) and water (Fig. 3D) consumption of db/db mice in the AL group were reduced in the SMF group. More importantly, SMF alone, IF alone, and SMF + IF all reduced the fasting blood glucose levels in db/db mice during the early stages of diabetes development (Fig. 3E and F). However, the effects of IF alone did last at the end of the third month (Fig. 3G to I) and were completely abolished at the end of the fourth month (Fig. 3J). In contrast, SMF combined with IF has a persistent and significant fasting blood glucose reduction effects throughout the whole experiment (Fig. 3F). At the end of the fourth month, the fasting blood glucose in the SMF + IF group is ~20 mM, while the AL group is ~30 mM, and IF group is ~30 mM (Fig. 3J). The reduction rate of SMF + IF is 34.4% compared with the AL group.

To get a more comprehensive assessment, we also examined glycosylated hemoglobin (HbA1c) (Fig. 3K) and glycated serum protein (GSP) (Fig. 3L) levels, which reflect the blood glucose control ability. For both HbA1c and GSP, SMF + IF group achieved better results than either SMF or IF alone. Moreover, the ITT also revealed that SMF + IF had the lowest blood glucose area under the curve (AUC) value (Fig. 3M and N), indicating better insulin sensitivity. In addition, the lifespan of db/db mice in the AL group could also be prolonged by SMF treatment (Fig. 3O and P). The average lifespan of db/db mice in the AL sham control group is 245 d, while the SMF-treated group was 310 d (*n* = 6 for each group). The lifespan of SMF is 26.5% longer than the AL group, but there was no statistical significance between the 2 groups due to the large difference in the survival time of mice within the groups.

### SMF + IF improves pancreatic islet and reduces complications including fatty liver in severe diabetic mice

**Fig. 4.**
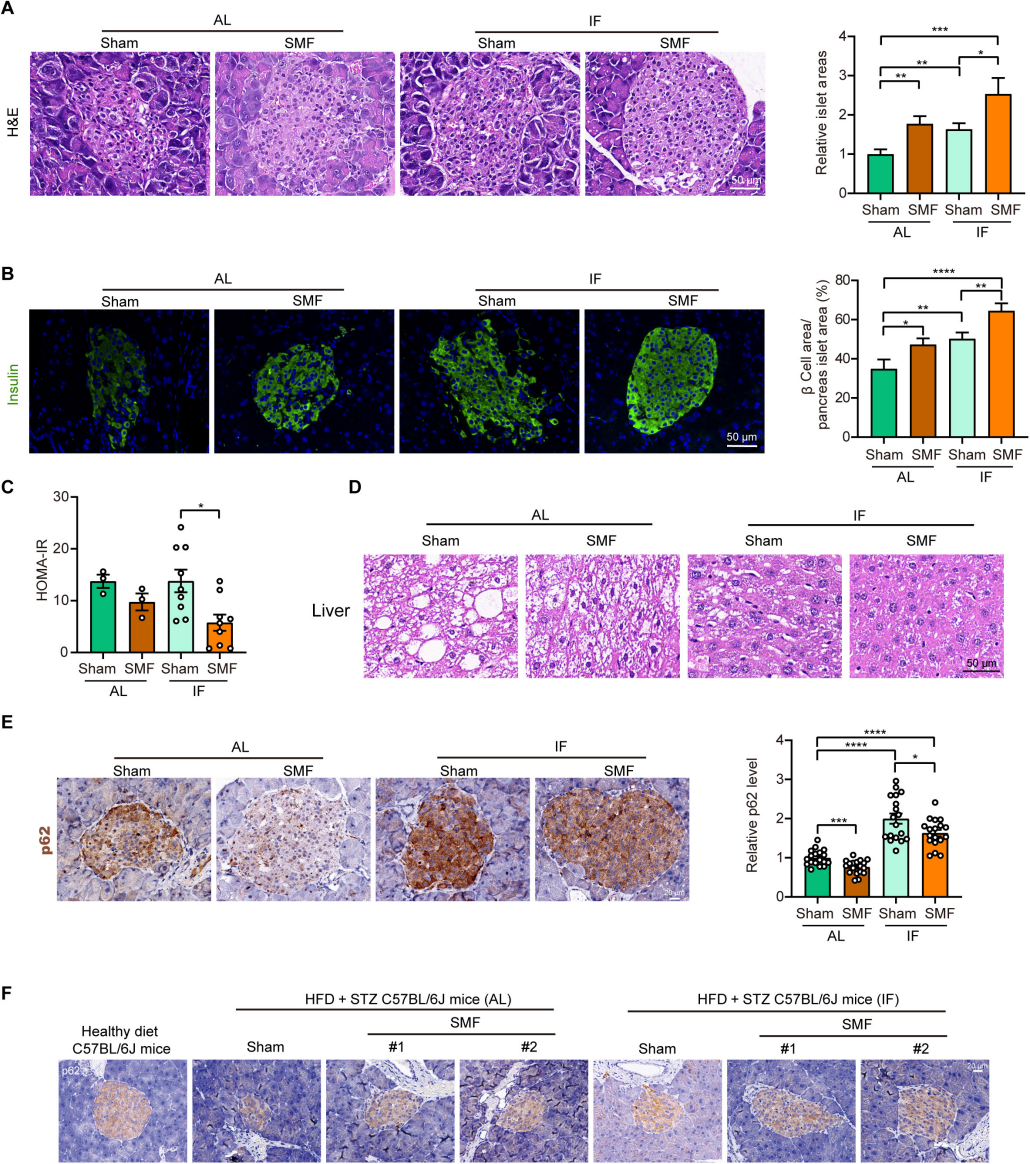
SMF combined with IF promotes pancreatic islet function in diabetic mice via increased autophagy. (A to E) Genetically diabetic BKS-Leprdb/J (db/db) mice were used. (A) Representative H&E images of mouse pancreatic islets and quantification of pancreatic islet area (*n* = 20 islets per group and 3 mice per group). (B) Representative immunofluorescence images of insulin in mouse pancreatic islets and quantification of β cell area (*n* = 12 to 18 islets per group and 3 mice per group). (C) HOMA-IR (*n* = 3 to 9 mice per group). (D) Representative H&E images of mouse liver (*n* = 3 mice per group). (E) Representative p62 immunohistochemical images and quantifications of db/db mouse pancreatic islets (*n* = 18 islets per group and 3 mice per group). (F) Representative LC3B immunohistochemical images of pancreatic islets from HFD + STZ-induced C57BL/6J mice (*n* = 3 mice per group). All data are presented as means ± SEM and analyzed by GraphPad Prism 9.0. *P < 0.05, **P < 0.01, ***P < 0.001, and ****P < 0.0001, 2-tailed Student’s *t* test (A to C and E).

Next, we evaluated the pancreatic islet quality in severe diabetic mice. We observed evident atrophy and vacuolation in the islets of db/db mice, while SMF + IF could significantly reverse this condition and increase pancreatic islet area (Fig. 4A). Immunofluorescence staining for insulin in pancreatic islet tissues reveals a significant increase in the β cell area in the SMF + IF group (Fig. 4B). These results are consistent with that of HFD + STZ-induced type 2 diabetes mice (Fig. 2E and F). Moreover, the HOMA-IR value indicates an improvement in insulin resistance in mice with SMF + IF (Fig. 4C).

Diabetic complications were also investigated. For example, we found that the fatty liver of db/db mice was significantly reduced by SMF and IF and completely prevented in SMF + IF (Fig. 4D and Fig. S4B). The organ index also indicates an improvement in the enlargement or pathology of db/db mouse liver and kidney after SMF or IF treatment (Fig. S4A). Furthermore, after SMF + IF treatment, there was a obvious amelioration in the vacuolization, irregular shape, and/or atrophy in the glomeruli and renal tubules of db/db mice. The irregular cell arrangement in the nuclear layer of retinal tissue was also alleviated, the disordered arrangement of hippocampal neuronal cells improved, and the arrangement of muscle cells became more compact (Fig. S4B). We also analyzed the mechanical withdrawal threshold by a Von Frey aesthesiometer because diabetic patients often experience complications related to neuropathy. The increased threshold in db/db mice suggested that SMF combined with IF might improve diabetic neuropathy (Fig. S4C). Moreover, behavior tests show that IF and SMF + IF have a tendency to improve the exercise and exploration ability of db/db diabetic mice (Fig. S5). Therefore, these results suggest that the SMF with IF can significantly alleviate diabetic complications.

### SMF + IF up-regulates autophagic levels in pancreatic islet cells of diabetic mice

To assess the cellular changes that contribute to improved pancreatic islet, we first examined apoptosis. We observed apoptosis in the islet β cells of db/db mice, which was reduced in SMF + IF group, as indicated by the reduced occurrence of terminal-deoxynucleotidyl-transferase-mediated deoxyuridine triphosphate nick end labeling-positive β cells (Fig. S6A). To inspect whether changes in pancreatic β cell proliferation contributed to the increase in pancreatic β cell proportion, we also examined Ki67, a marker of cell proliferation. We found very low levels of β cell proliferation in all groups across both batches of diabetic mice (Fig. S6B). We also checked apoptosis and Ki67 of HFD + STZ-induced C57BL/6J diabetic mice but did not reveal significant differences among the experimental groups (Fig. S6C and D). These results indicate that apoptosis and proliferation are not the major reason for improved pancreatic islet of SMF + IF in diabetic mice.

Previous studies have indicated a protective role of autophagy in pancreatic β cells [26–28], but autophagy was also found to be impaired in both type 1 and type 2 diabetes [29,30]. Since IF can promote autophagy [5,8,31], we next investigated whether long-term treatment of SMF and/or IF can exert their beneficial effects this way. At the end of 4-month treatment, we performed immunohistochemical staining for LC3B (autophagosome marker) and p62 (autophagy receptor and substrate) in the pancreatic islet tissues of diabetic mice (Fig. 4E and Fig. S7). In the pancreatic islet slices of both types of type 2 diabetes mice, SMF + IF increased LC3 levels (Fig. S7A, C, and D). The IF treatment evidently increased p62 amount compared with the AL group in severe db/db mice (Fig. 4E), but not in the HFD + STZ-induced moderate type 2 diabetes mice (Fig. 4F and Fig. S7B). In the presence of SMF, the p62 decreased in both AL and IF groups (Fig. 4E). Since severe db/db mice have much higher blood glucose levels than moderate HFD + STZ-induced type 2 diabetes mice (30.08 ± 1.01 mM versus 17.96 ± 1.34 mM) and autophagy has been reported to be impaired in diabetes [29,30], we think that the p62 accumulation in IF-treated mice is a sign for decreased autophagic flux because p62 not only serves as both autophagy receptor or adapter that is up-regulated by autophagy but also serves as substrate that is degraded by autophagy.

### SMF promotes IF-induced hepatic autophagy levels and improves liver metabolism in diabetic mice

**Fig. 5.**
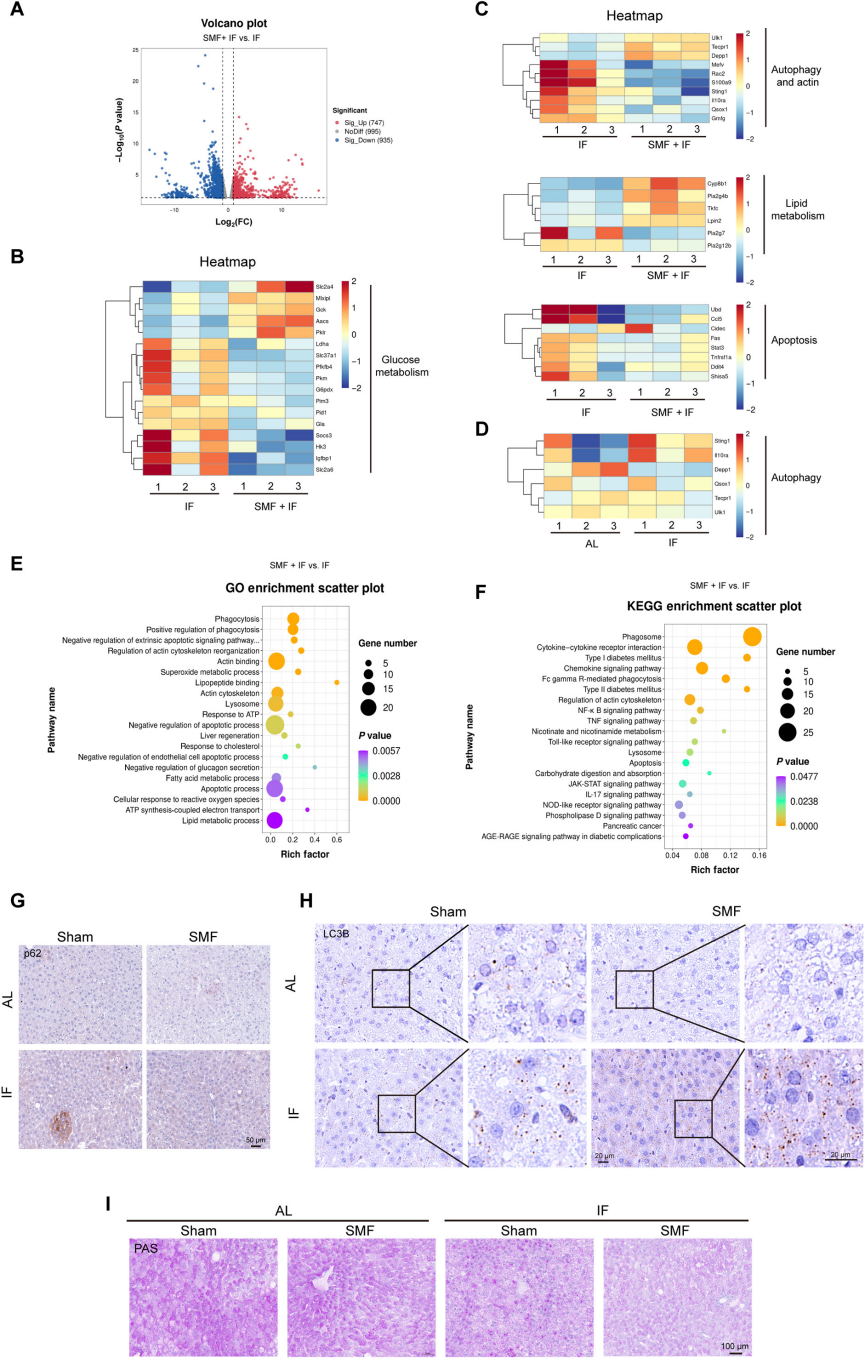
SMF combined with IF promotes hepatic autophagy level and improves liver metabolism in severe db/db diabetic mice. (A to C) Volcano plot (A), heat map (B and C) of the differential expression genes from SMF + IF versus IF mice. (D) Heatmap of the differential expression genes from IF versus AL mice. (E and F) GO (E) and KEGG (F) terms of the differential expression genes from SMF + IF versus IF mice. (G to I) Representative p62 (G) and LC3B (H) immunohistochemical images and PAS images (I) of liver from db/db mice (*n* = 3 mice per group). FC, fold change; ATP, adenosine 5′-triphosphate; NF-κB, nuclear factor κB; TNF, tumor necrosis factor; JAK, Janus kinase, STAT, signal transducers and activators of transcription; IL-17, interleukin-17; NOD, nonobese diabetic; AGE-RAGE, advanced glycation end product-receptor for advanced glycation end products.

It is well known that IF has been demonstrated as an effective way to improve diabetes and related complications. To get a comprehensive understanding on the effects of SMF, we conducted RNA sequencing (RNA-seq) analysis to compare the mouse liver tissues of IF and SMF + IF. When using a threshold of *P* < 0.05 and |log_2_(fold change)| > 1, we found 747 up-regulated and 935 down-regulated genes (Fig. 5A). Further analysis revealed significant differences in the expression levels of genes related not only to glucose metabolism (such as *Sla2a4*, *Aacs*, *Pim3*, and *Igfbp1*; Fig. 5B) but also in autophagy and actin-related genes (such as *ULK1*, *Dram1*, *S100a9*, and *Gmfg*; Fig. 5C), lipid metabolism-related genes (such as *Cyp8b1* and* Tkfc*), and apoptosis-related genes (such as *Fas*, *Tnfrsf1a*, etc.). More specifically, when compared to individual IF treatment, genes positively correlated with autophagy such as *ULK1*, *Tecrp1*, and *Depp1 *were up-regulated in the SMF + IF, while genes negatively correlated with autophagy, such as *Sting1*,* Il10ra*, and *Qsox1*, were down-regulated under SMF + IF conditions. This confirms the autophagy-promoting function of SMF on the basis of IF stimulation. However, when the individual IF group was compared to the AL group, the changes in these genes did not exhibit a trend toward promoting autophagy (Fig. 5D), which indicates that the autophagy levels are not increased by IF at this stage. In addition, Gene Ontology (GO) pathway enrichment (Fig. 5E) and Kyoto Encyclopedia of Genes and Genomes (KEGG) pathway enrichment (Fig. 5F) were used to evaluate the pathways enriched by these differentially expressed genes. GO terms indicate that the differentially expressed genes were closely associated with processes such as cell apoptosis, actin cytoskeleton, lipid metabolism, glucose metabolism, and autophagy. KEGG terms further confirm the involvement of these differentially expressed genes in autophagy, cell apoptosis, and metabolism-related pathways.

Based on the RNA-seq results, we performed immunohistochemical staining for LC3B, p62, and PAS (periodic acid-Schiff) glycogen in the liver tissues of db/db mice (Fig. 5G to I). We found that IF or SMF + IF can significantly increase the p62 and LC3B level in the liver tissues and the LC3B changes were more obvious in SMF + IF (Fig. S8A and B). Furthermore, PAS staining revealed that SMF + IF can reduce hepatic glycogen accumulations significantly (Fig. S8C). As a result, the liver’s ability to absorb the next dose of glucose and store it as glycogen is enhanced under lower-glycogen conditions, thereby improving glucose metabolism.

Besides, we noticed that the RNA-seq results showed GO analysis enrichment in the cellular response to reactive oxygen species (ROS). Our previous research also indicated that SMF treatment could reduce cellular oxidative stress [17,32]. Therefore, we analyzed oxidative-stress-related indicators here. First, we compared the differentially expressed genes between the IF and SMF + IF groups and found some oxidative-stress-related genes, such as *Mpo*, *Fpr2*, *Ncf1*, and *Ncf2* (Fig. S9A). Furthermore, we examined oxidative stress indicators in the liver tissue of db/db mice and found that both SMF and SMF + IF significantly reduced ROS levels and nuclear factor erythroid 2-related factor 2 (NRF2) levels in the liver (Fig. S9B to E). Interestingly, we found that although SMF could also reduce ROS in mouse islet tissue, the overall ROS level in islets was relatively low, especially compared to the surrounding pancreatic tissue (Fig. S9F and G). The NRF2 levels in the islets were also low, with no differences between the groups (Fig. S9H). This may be related to the lower expression levels of antioxidant enzymes in islet cells [33,34], making them more sensitive to oxidative stress. Consequently, their ROS levels are typically maintained at a lower state to prevent oxidative damage. On this basis, we believe that oxidative stress might not be the key mechanism through which islets are protected and the changes in autophagy may play a more important role.

### SMF + IF up-regulates autophagic flux of pancreatic islet cells in vitro

**Fig. 6.**
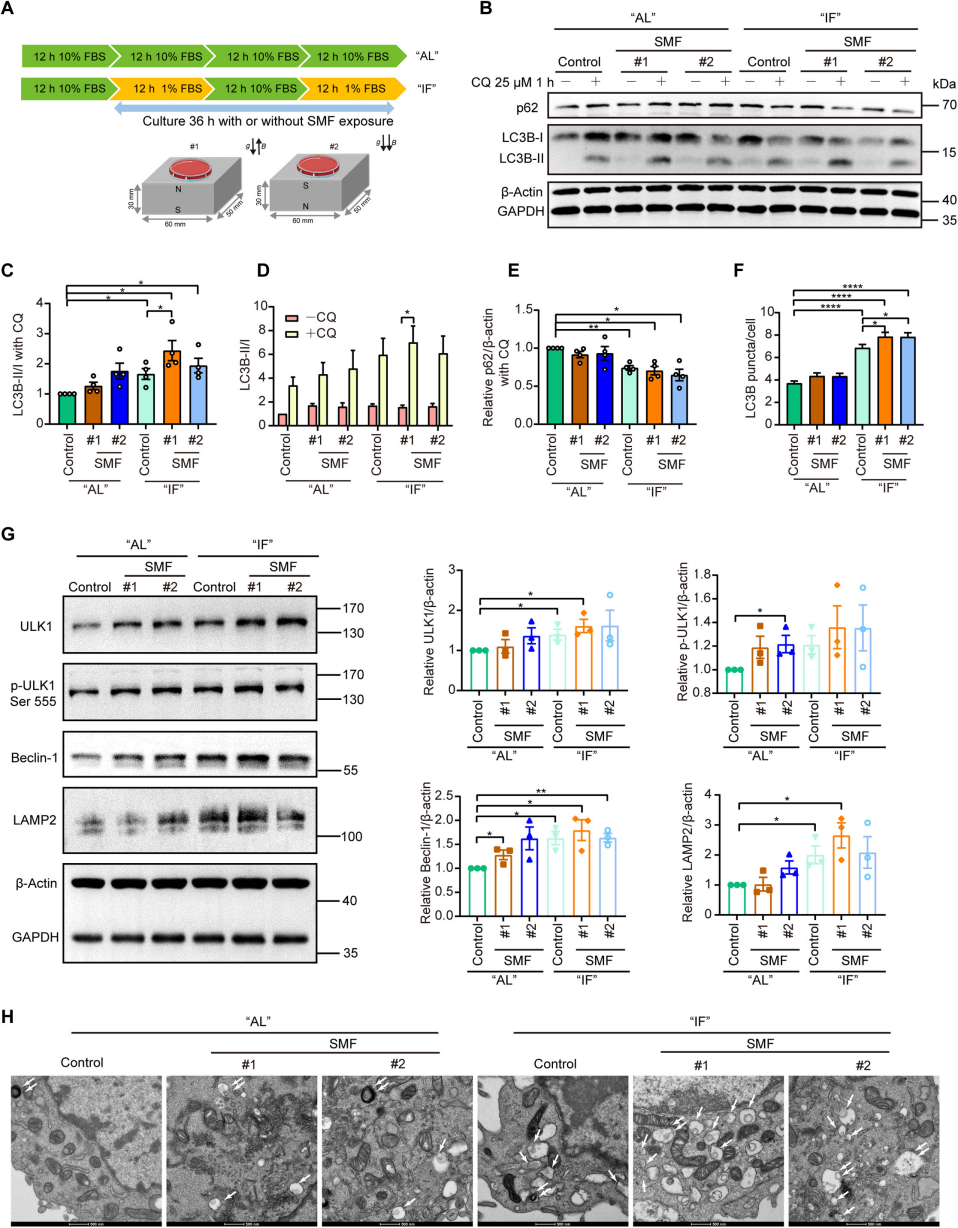
SMFs promote IF-induced autophagy levels in Min6 cells in vitro. (A) Cellular experimental setup and design. (B to H) Intermittent starvation of Min6 cells using 1% serum starvation was carried out for 48 h with simultaneous magnetic fields treatment for 36 h. (B) Min6 cells were treated with or without autophagy inhibitor 25 μM CQ for 1 h. Representative western blot images for p62 and LC3B. (C to E) Quantification of LC3B (C and D) and p62 (E) from (B). (F) LC3B puncta number of Min6 cell immunofluorescence image (see also Fig. S10A, count 200 cells per group). (G) Representative Western blot images and quantification of autophagy pathway markers. (H) Representative electron microscopy images of autophagic structures of Min6 cells. (The white single arrows represent autophagosomes, and the white double arrows represent autolysosomes.) Scale bars, 500 nm. All data are presented as means ± SEM and analyzed by GraphPad Prism 9.0. *P < 0.05, **P < 0.01, and ****P < 0.0001, 2-tailed Student’s *t* test (C to G). FBS, fetal bovine serum; N, north; S, south;

In fact, LC3 and p62 protein levels at a fixed time point cannot accurately reflect the exact autophagic flux levels. For example, p62 is not only an autophagy receptor but is also an autophagy substrate. Increased p62 can indicate up-regulated autophagy pathway or decreased autophagic flux, which lead to p62 accumulation. Therefore, to get an accurate measurement of the autophagy states, we further designed in vitro cellular experiments to mimic the mouse “AL” and “IF” feeding patterns using mouse pancreatic β cell line Min6 cells. The cells were subjected to 1% serum starvation instead of the regular 10% serum to mimic “IF”, and the SMF treatment time is 36 h (Fig. 6A). We measured autophagic flux using chloroquine (CQ), which blocks the degradation of LC3B-II and leads to its accumulation (Fig. 6B). We found that IF significantly increased the LC3B-II/I ratio and reduced p62 levels in Min6 cells and the effect was more pronounced in SMF + IF, indicating higher autophagy levels (Fig. 6C to E). To further confirm this phenomenon, we also counted the number of punctate LC3B in Min6 cells under a microscope, which also confirms that SMF + IF increases cellular autophagy levels (Fig. 6F and Fig. S10A). Moreover, we also examined the levels of key autophagy pathway proteins in Min6 cells. Unc-51-like autophagy activating kinase 1 (ULK1) is involved in autophagosome formation, Beclin-1 serves as an autophagic initiator, and lysosome-associated membrane protein 2 (LAMP2) regulates the lysosome-autophagosome fusion process. The results showed that SMF + IF significantly up-regulated the expression levels of ULK1, p-ULK1 Ser555, Beclin-1, and LAMP2 (Fig. 6G). Furthermore, using transmission electron microscopy, we confirmed that the increased numbers of autophagosomes and autolysosomes by SMF and IF are further increased by SMF + IF (Fig. 6H). These experimental results confirm that SMF can up-regulate autophagic flux in pancreatic Min6 cells.

### SMF enhances IF-induced autophagy by promoting actin polymerization

**Fig. 7.**
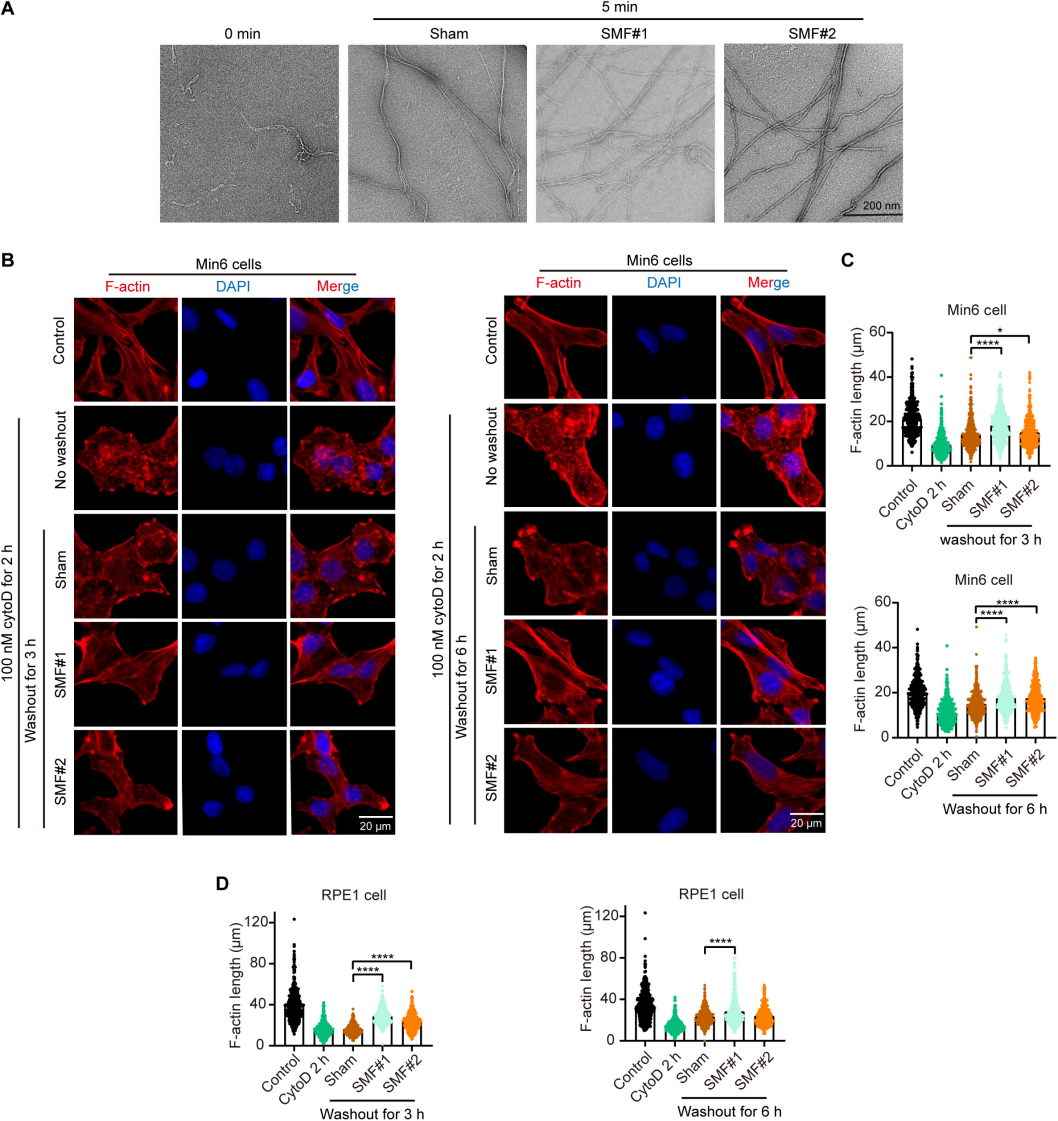
Actin assembly is directly affected by magnetic fields. (A) Representative transmission electron microscopy images of F-actin assembled in 0 min, sham in 5 min, SMF#1 in 5 min, or SMF#2 in 5 min, in the regular geomagnetic field environment in the laboratory (~0.5 to 0.8 Gs). Scale bar, 200 nm. (B) Min6 cells were treated with 100 nM cytoD for 2 h, with or without additional washout to allow recovery for 3 or 6 h with sham or SMF, before they were fixed and stained with phalloidin and 4′,6-diamidino-2-phenylindole (DAPI). Scale bars, 20 μm. (C) Quantification of F-actin filament length from Min6 cell (the length of 500 F-actin filaments was measured). (D) Quantification of F-actin filament length from RPE1 cells (the length of 500 F-actin filaments was measured). All data are presented as means ± SEM and analyzed by GraphPad Prism 9.0. *P < 0.05 and ****P < 0.0001, 2-tailed Student’s *t* test (C and D).

It is well known that the generation of autophagosomes involves membrane rearrangement and movement, a process for which the actin cytoskeleton provides dynamic support [35–37]. We conducted in vitro actin polymerization assays and found that after only 5 min of treatment with SMF, actin exhibited an increased formation of fibrous structures (Fig. 7A), indicating that SMFs can promote actin polymerization. To test the effects of SMF in cells, we treated the Min6 cells with cytochalasin D (cytoD) for 2 h to induce actin depolymerization and observe its recovery by drug washout with or without SMF treatment for 3 or 6 h (Fig. 7B and C). The actin polymer formation is increased in the SMF-treated groups compared with the sham control group. We also confirmed this phenomenon in retinal pigment epithelium 1 (RPE1) cells (Fig. 7D and Fig. S10B and C). These results confirm our hypothesis that SMF can enhance IF-induced autophagy by promoting actin polymerization in cells.

## Discussion

**Fig. 8.**
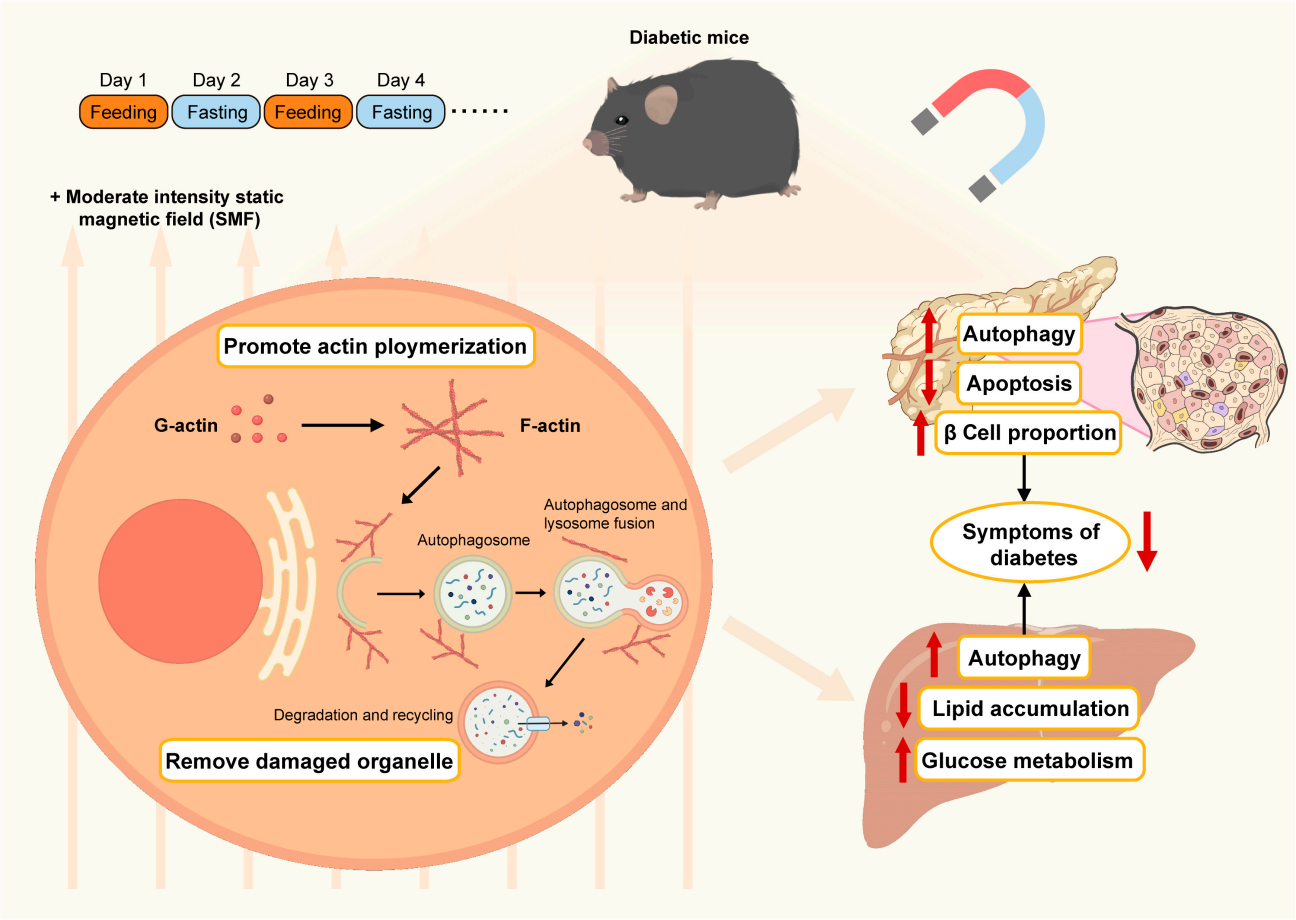
Mechanism of SMFs combined with IF in diabetic mice.

Autophagy is known to play a crucial role in clearing damaged organelles and protecting cells. Our study shows that the combination of moderate SMF with IF leads to a significant alleviation of severe type 2 diabetes, surpassing the effects of IF alone. In vivo and in vitro evidences show that this enhancement could be attributed to actin polymerization promotion by SMF, which further augments IF-induced autophagy, leading to improved pancreatic β cell and liver metabolism (Fig. 8).

Our results show that the combination of SMF and IF is very effective on both diabetic mouse models. In the moderate diabetic mice, the fasting blood glucose level is 17.96 ± 1.34 mM, which is significantly higher than the healthy control (8.3 ± 0.28 mM). However, the SMF + IF can lower the fasting blood glucose level to 10.68 ± 0.93 mM, which is much better than the IF alone (15.19 ± 1.17 mM) and very close to the healthy control. In the severe diabetic mice, by monitoring the progression for 4 months, we found that IF do have beneficial effects on diabetes by effectively decreasing the blood glucose levels, which is consistent with the literature. However, the beneficial effects of IF gradually diminish at the later stages. Specifically, IF can lower the fasting blood glucose by 43.7%, 42.2%, and 23.5% at the end of the first, second, and third months, respectively. In contrast, by the end of fourth month, IF has no effect at all. In other words, the severe diabetic mice become “IF-resistant” at later stage, when the fasting blood glucose level exceeds 30 mM. In contrast, SMF + IF has a persistent effect on reducing fasting blood glucose level, which is 34.4 % lower than the sham untreated group (19.73 ± 2.72 mM versus 30.08 ± 1.01 mM, *P* < 0.05) and 36.2% lower than IF group (19.73 ± 2.72 mM versus 30.92 ± 0.84 mM, *P* < 0.01) at the end of 4 months. These results demonstrate that severe diabetic mice can benefit from IF alone at the beginning but become “IF-resistant” at later stages. In contrast, SMF + IF has a consistent blood glucose lowering effects in severe diabetic mice.

In addition, we noticed that the “IF-resistant” in HFD + STZ-induced moderate diabetic mice is not as evident as in the db/db severe diabetic mice. This may be attributed not only to the severity of diabetes but also to the overall duration of fasting. For the db/db severe diabetic mice, the whole experiment was much longer, in which we performed IF for 120 d and the AL group has fasting blood glucose of 30.08 ± 1.01 mM. For HFD + STZ moderate diabetic mice, the IF treatment was for 6 weeks, and the AL group has fasting blood glucose of 17.96 ± 1.34 mM. It seems that longer time of IF and high blood glucose levels lead to “IF-resistant” phenomenon. We think that the prolonged exposure of diabetic islets to high-glucose environment has led to glucotoxicity and impaired autophagic flux in pancreatic β cells, which results in p62 accumulation. In this case, SMF could facilitate the restoration of autophagic flux, leading to reduced p62 accumulation and improved islet.

Autophagy is the key cellular process that contributes to the beneficial effects of SMF + IF on diabetic mice. Pancreatic β cells exposed to prolonged high-glucose and high-lipid stimuli have increased protein ubiquitination and damaged organelle accumulation. If not promptly cleared, toxic aggregates can form and induce β cell apoptosis [28]. Autophagy can protect β cells from apoptosis by timely clearing dysfunctional organelles [26,27]. Similarly, liver cells are also affected by prolonged glycotoxicity and lipotoxicity, leading to metabolic disorders [38]. In this context, autophagy plays a protective role by reducing liver lipid accumulation and improving liver metabolism [39]. For the db/db mice after prolonged treatment of IF and/or SMF for 4 months, we found that IF group has p62 accumulation, while SMF leads to p62 reduction in pancreatic islet. Autophagic flux analysis using Min6 cells and RNA-seq analysis of liver tissues show that the autophagic flux level is increased in the SMF + IF. However, at this late diabetic stage, the autophagic flux level in the IF alone is lowered, which leads to p62 degradation block. At the end of our experiment, liver RNA-seq was conducted on db/db mice. The results revealed that, compared to the AL group, the genes positively correlated with autophagy were not up-regulated in the IF group alone and the genes negatively correlated with autophagy were not down-regulated either. This suggests that during the late stages of diabetes, the autophagic flux induced by IF is gradually diminishing.

Actin cytoskeleton dynamics are known to play vital roles in autophagy. It has been shown that actin polymerization is necessary for the biogenesis of autophagosomes from the endoplasmic reticulum membrane, structurally supporting the expanding phagophore and the later steps of autophagosome-lysosome fusion [37,40]. Promoting actin polymerization has been shown to work effectively to increase autophagy [41]. More importantly, the actin cytoskeleton plays important roles in the early events of autophagosome formation upon starvation-induced autophagy [35]. It has been shown that annexin A2 is an autophagy regulator that regulates autophagosome formation by transporting autophagy-related 9A (ATG9A) from the endosome to the autophagosome via actin [42]. Moreover, it is interesting that when the actin cytoskeleton is depolymerized, the increase in autophagic vacuoles in response to the starvation stimulus was abolished without affecting maturation of remaining autophagosomes. Since glucose can induce F-actin remodeling in pancreatic β cells [43] and diabetes can induce F-actin spatial organization [44], we hypothesize that the altered actin cytoskeleton dynamics may be the reason for the later-stage “IF resistance” in severe diabetes. In this case, the application of SMF, which can boost actin polymerization, can efficiently increase autophagy to improve pancreatic β cell. Previous studies have reported that rotating magnetic fields can affect actin related genes [45] or directly perturb F-actin [46], and our findings further confirm the direct effect of magnetic fields on actin dynamics.

It should be mentioned that SMF alone is also effective in some aspects, although much weaker than the SMF + IF. In fact, we did a preliminary pilot study to test the survival of the db/db mice since their diabetes is progressively severe and detrimental. Because of the long duration of the experimental design (~4 months) and the limitation of mouse space in the animal facility, we only tested a small number of mice, which is an obvious limitation of our study. However, it is interesting SMF alone can increase the lifespan of db/db mice from 245 to 310 d. This is actually consistent with the multiple evidences about the alleviating effects of SMF on the mouse diabetic symptoms, including improved pancreatic islet and liver. SMF also reduces the food and water intake of db/db mice, indicating improvement of diabetic mouse hyperphagic state. Future experiments using larger sample size, lifelong treatment, and more mouse models with SMF alone with/without IF will be necessary to get a complete assessment of the effectiveness of these nonpharmacological strategies on the progression and final outcomes of diabetes, as well as other conditions, such as obesity.

It is worth mentioning that our previous study has found the beneficial effects of moderate SMFs on type 2 diabetes mellitus [17]. In that study, we used near-homogeneous SMF provided by neodymium magnetic plate with the highest SMF intensity we could get. However, that device is not only expensive but also hard to fabricate, heavy, and dangerous to move around. Therefore, here, we tried to use a different strategy using a combination of multiple cylindrical permanent magnets with a surface magnetic flux density of 0.5 T, which is also the highest intensity we could get. In this case, the cost and weight of the device are both reduced by ~10-fold, and it is safe to move around. In addition, we also compared the potential “preventive” and “therapeutic” effects of SMFs by applying SMFs before or after the initiation of diabetes and found that both methods worked.

In conclusion, our study demonstrates that the combination of moderate-intensity SMF with IF can significantly improve type 2 diabetes by increasing the levels of autophagy in the pancreatic islet and liver. The “IF-resistant” phenomenon of severe diabetic mice at later stage can be reversed. In fact, this combination of physical therapy and dietary intervention not only avoids the toxic side effects associated with drug treatments but also offers the advantages of being noninvasive, cost-effective, and practical. Therefore, our study provides insights for a new antidiabetic strategy, which may be valuable for patients with severe diabetes but are poorly controlled by conventional drugs.

## Materials and Methods

### Magnetic fields setup

For animal experiments, we used 8 cylindrical neodymium iron boron (NdFeB) N38 permanent magnets (diameter × height: 16 mm × 7 mm) embedded in a polyvinyl chloride board as a unit (length × width: 120 mm × 60 mm), with a surface maximum magnetic flux density of 0.5 T. We fixed 10 units on an acrylic plate. We have 3 sets in total, one with the north pole facing up (SMF#1), one with the south pole facing up (SMF#2), and one with unmagnetized NdFeB as “sham” control. The mouse cages were directly placed on the top of these magnetized or unmagnetized plate (length × width: 310 mm × 250 mm) and treated for 24 h/d.

For cellular experiments, we used NdFeB N38 permanent magnets (length × width × height: 60 mm × 50 mm × 30 mm) with a surface maximum magnetic flux density of 0.5 T. The magnets were placed inside the cell culture incubator, which was maintained at a temperature of 37 °C with 5% CO_2_. The cell culture dishes were placed above the north or south pole of the magnets (SMF#1 and SMF#2). The control group was placed inside the same cell culture incubator but far away from the magnets. The SMF of control group is ~0.0001 T, which is 5,000 times less than the SMF group (Fig. S11).

A magnetic field analyzer (FE-2100RD, Hunan Forever Elegance Technology, China) was used to measure the magnetic flux density distribution of all magnetic devices used in this study.

### Animal models

We used 2 different mouse models in this study, representing moderate diabetes (HFD + STZ-induced, fasting blood glucose levels is ~20 mM) versus severe diabetes (genetically mutant db/db mice, fasting blood glucose levels is higher than 30 mM at later stage).

Four-week-old male BKS-Leprdb/J (db/db) and C57BL/6J mice from the Nanjing Biomedical Research Institute of Nanjing University (Nanjing, China) were housed in barrier environment air-conditioned rooms at 22 to 24 °C and 50 to 60% humidity, with a 12-h light-dark cycle. There were 89 C57BL/6J mice in total, including 9 mice in the control group and 10 mice in each other group. Mice that were not successfully induced diabetes were excluded, and the final numbers of each group were 8 to 9. For db/db mice, we used 54 mice and randomly divided them into 6 groups (9 mice in each group). They had AL access to food and water. After 1 week of adaptation, the control group of C57BL/6J mice was fed a regular diet, while the other groups were given an HFD (60 kcal % of fat, D12492, Research Diet Company, USA; Table S1). When the mice were 11 weeks old, the sham group was treated with a buffer solution, while the experimental groups received intraperitoneal injections of STZ at a dose of 45 mg/kg in 0.01 M citrate buffer for 4 consecutive days.

All in vivo experiments complied with the guidelines approved by the Animal Protection and Utilization Committee of the Hefei Institutes of Physical Science, Chinese Academy of Sciences for all protocols (approval no. DWLL-2022-10).

### Intermittent fasting

The mice were fed with chow or HFD, and the IF procedure was performed as reported [9]. Briefly, mice were allowed to eat AL for 24 h and then fasted for 24 h as a cycle for the experiment. During fasting, the mice were completely deprived of food, but had unrestricted access to water. Throughout the study, the food intake and water consumption were recorded. The average food intake and water consumption per mouse were calculated. The mice were weighed every 7 to 8 d on the feeding days.

### Fasting blood glucose level, intraperitoneal ITTs and intraperitoneal GTT

For fasting blood glucose level measurement along the IF experiments, both batches of mice were fasted for 6 h. During HFD + STZ induction period in C57BL/6J mice, according to the literature [17,47], the mice were fasted overnight to examine whether their fasting blood glucose levels have reached the standard threshold for diabetic mice.

We conducted intraperitoneal GTTs (IPGTTs) and intraperitoneal ITTs (IPITTs) experiments 1 week before the end of mouse experiment. Both IPGTT and IPITT were performed on the fasting day. For IPGTT, mice were fasted for 6 h before their blood samples were collected through the tail vein, and blood glucose concentrations were measured using a glucose meter. After intraperitoneal injection of glucose (0.75 g/kg), glucose measurements were performed at 0, 30, 60, 90, and 120 min after injection. For IPITT, mice were fasted for 4 h before intraperitoneal injection of insulin (0.75 U/kg). Their blood glucose levels were measured at 0, 30, 60, 90, and 120 min after injection. The glucose AUC during the IPGTT process was calculated using the trapezoidal rule.

### RNA-seq analysis

Liver tissues from db/db mice were collected, and total RNA was extracted using TRIzol reagent (Thermo Fisher Scientific) following the manufacturer’s protocol. The quantity and purity of total RNA were analyzed using the Bioanalyzer 2100 and RNA 6000 Nano LabChip Kit (Agilent). Subsequently, the fragmented RNA was reverse-transcribed using SuperScript II reverse transcriptase (Invitrogen). Then, U-labeled second-stranded DNA was synthesized using *Escherichia coli* DNA polymerase I [New England Biolabs (NEB)], ribonuclease H (NEB), and deoxynucleotidyl transferase solution (Thermo Fisher Scientific). Next, an A-base was added to the blunt ends of each strand, preparing them for connection to index adapters. U-labeled second-stranded DNA was treated with heat-labile uracil-DNA glycosylase (UDG) enzyme (NEB), and the resulting connected products were amplified by polymerase chain reaction. Finally, 2× 150-base-pair paired-end sequencing (PE150) was performed using Illumina NovaSeq 6000 (LC-Bio Technology Co. Ltd., Hangzhou, China). Bioinformatic analyses, including volcano plot, heatmaps, and GO and KEGG pathways, were conducted using OmicStudio tools available at https://www.omicstudio.cn/tool.

### Statistical analysis

The experimental data are presented as means ± SEM and analyzed using the unpaired or paired 2-tailed Student’s *t* test. Statistical analysis was performed using GraphPad Prism 9.0 software (GraphPad Software Inc., La Jolla, CA, USA, RRID:SCR_002798), and *P* < 0.05 was considered statistically significant.

## Data Availability

All data needed to evaluate the conclusions in the paper are present in the paper and/or the Supplementary Materials. Additional data related to this paper may be requested from the authors.
