# Supplementary material for: Enhanced Effects of Intermittent Fasting by Magnetic Fields in Severe Diabetes
Source: Research (Wash D C). 2024 Sep 5;7:0468. doi: 10.34133/research.0468 (PMC11376831; doi:10.34133/research.0468)
Supplement: Supplementary 1 — Supplementary Methods Figs. S1 to S11 Table S1 [file research.0468.f1.pdf]

Supplementary Materials for

Enhanced effects of intermittent fasting by magnetic field in severe diabetes

Ying Wang[1,2,†], Chuanlin Feng[1,2†], Biao Yu[1,3], Junjun Wang[1], Weili Chen[1,4], Chao Song[1], Xinmiao Ji[1,2], Ruowen Guo[1,2], Guofeng Cheng[1,2], Hanxiao Chen[1,2], Xinyu Wang[1,4], Lei Zhang[1], Zhiyuan Li[5], Jialiang Jiang[1,2], Can Xie[1,2], Haifeng Du[1,2,4], Xin Zhang[1,2,4]

Correspondence to: xinzhang@hmfl.ac.cn

This PDF file includes:

Supplementary Methods
Fig. S1 to S11
Tables S1

Supplementary Methods

Cell culture

Min6 cell (RRID: CVCL_0431) and RPE1 cell (RRID: CVCL_4388) were obtained from the American Type Culture Collection (ATCC, Manassas, VA, USA) and cultured in DMEM (15-017-CV, Corning, Manassas, USA) containing 10% fetal bovine serum and 1% penicillin/streptomycin. The cells were incubated at 37 °C and 5% CO_2 in a cell incubator (Thermo Fisher Scientific). For the IF experiment with the cells, after 12 h of adherent culture in nutrient-rich medium, the cells were washed and subjected to two 12-h periods of starvation by culturing them in DMEM containing 1% FBS. In between the fasting periods, the cells were cultured in DMEM containing 10% FBS for 12 h.

Serum test

At the end of the experiment, the mice were sacrificed to collect blood samples. Immediately after collection, the serum was obtained by centrifuging the blood samples at 4000 × g for 10 minutes. GSP, ALT, AST, TG, CHO, HDL and LDL, were analyzed using a Chemray 240 fully automatic biochemical analyzer (Rayto Corporation, Shenzhen, China). The levels of glycated hemoglobin in the mice's blood were measured using a glycated hemoglobin testing kit (A1CNow+, Sinocare, Changsha, China). The insulin levels in mice serum were measured using an insulin ELISA kit (m1001983-J, Mibio, Shanghai, China) following the manufacturer's instructions. The calculation formula for the homeostatic model assessment of insulin resistance (HOMA-IR) is as follows: fasting insulin level (FINS, μU/mL) ×fasting plasma glucose level (FPG, mmol/L) /22.5.

Histological analysis

At the end of the experiment, all mice were euthanized under anesthesia, and their hearts, livers, spleens, lungs, kidneys, pancreas, and other organs were collected. The samples were fixed in 4% neutral buffered formalin (Servicebio, Wuhan, China). After embedding in paraffin, 5 μm-thick sections were cut and stained with hematoxylin and eosin (H&E) and periodic acid-Schiff (PAS).

Immunohistochemistry

Immunohistochemical staining was performed on mice pancreatic and liver tissues, following the manufacturer's instructions for all steps. The antibodies used included Ki67 (Abcam Cat# ab15580, RRID: AB_443209), LC3B (Abcam Cat# ab192890, RRID: AB_2827794), p62 (Abcam Cat# ab109012, RRID: AB_2810880), NRF2 (Servicebio Cat# GB113808-100). Complete images were obtained through scanning.

Immunofluorescence staining

Immunofluorescence staining for insulin was performed on mice pancreatic tissue, following the manufacturer's instructions for all steps. The antibody used was insulin (Abcam Cat# ab181547, RRID: AB_2716761). Complete images were obtained through scanning. Image J software (RRID:SCR_003070) was used for quantitative analysis of the microscopic regions. The insulin-positive area representing the insulin-positive cell population was used to quantify the positive area of pancreatic β-cells.

Similar immunofluorescence staining was performed for apoptosis marker TUNEL and insulin. The TUNEL assay was used to detect apoptotic cells in pancreatic tissues using the TUNEL detection kit (C1098, Beyotime, China), which labels dUTP with a nickel end. 3,3'-diaminobenzidine (DAB) reagent was used for staining, and the samples were incubated at room temperature for 5-30 minutes. Image J software was used to count TUNEL-positive cells in the insulin-labeled regions and quantitatively analyze the number of apoptotic β-cells in each islet.

DHE staining

DHE was dissolved in DMSO to detect ROS in mice liver and islets. Paraffin sections of liver and pancreatic islets after dewaxing were taken and treated with PBS and tissue autofluorescence quencher for 10 minutes. Then, 100 μl of staining working solution (DHE diluted at a ratio of 1:1000) was added, and the sections were incubated in the dark at 37 °C for 60 minutes. Finally, the sections were stained with 4',6-diamidino-2-phenylindole (DAPI) and sealed with an anti-fluorescence quencher.

Cell immunofluorescence

The Min6 cell treatment was performed as described above. Cells attached to coverslips were washed with PBS and fixed with -20 °C methanol for 5 minutes. After blocking the cells with Abdil-TX (0.1% Triton X-100, 2% bovine serum albumin, 0.05% NaN_3) for 30 minutes, immunofluorescence staining was conducted using LC3B (Abcam Cat# ab192890, RRID: AB_2827794) antibody and Alexa-488 conjugated anti-rabbit IgG. Subsequently, the cells were stained with 300 nM 4',6-diamidino-2-phenylindole (DAPI)

at room temperature for 5 minutes and then fixed with Antifade Pro-Long Gold (P36980, Invitrogen).

Both Min6 and RPE1 cells were seeded on 35 mm culture dishes containing coverslips and allowed to adhere for 24 h. After treating the cells with 100 nM cytochalasin D (cytoD) for 2 h, the culture medium was removed, and the cells were washed with PBS before changing to normal culture medium. Samples were collected at 3 h and 6 h after treatment. The cells on coverslips were washed with PBS, fixed with 4% paraformaldehyde at room temperature for 20 minutes, and then blocked with Abdil-TX (0.1% Triton X-100, 2% bovine serum albumin, 0.05% NaN_3) for 30 minutes. F-actin was stained with Alexa Fluor™594 phalloidin (A12381, Invitrogen) at room temperature for 0.5 h. Next, the cells were stained with 300 nM 4',6-diamidino-2-phenylindole (DAPI) for 5 minutes at room temperature, followed by fixing with Antifade Pro-Long Gold (P36980, Invitrogen). The images were captured using an Olympus fluorescence microscope (SpinSR10, Olympus, Tokyo, Japan). The length of F-actin filaments in cells were measured using Image J software.

Western blotting

Min6 cells (3×10^5 cells/mL) were seeded into 35 mm culture dishes and allowed to adhere to the walls. Subsequently, the cells were subjected to either regular culture or intermittent serum starvation. Simultaneously, they were exposed to sham, SMF#1 or SMF#2 for 36 h. To assess autophagic flux, some cell groups were treated with 25 μM CQ for 1 h. Cell lysates were prepared using lysis buffer containing inhibitors to extract proteins from Min6 cells for Western blot analysis. All proteins were separated by 8%-15% SDS-PAGE and

then transferred onto PVDF membranes. The membranes were blocked with 5% skim milk and incubated with different primary antibodies overnight at 4 ℃, followed by incubation with corresponding secondary antibodies at room temperature for 1 h. Western blotting results were visualized using the Bio-Rad ChemiDoc TM XRS+ system and Beijing Tanon Fine-do X6. Image J software was used to quantify the relative levels of the proteins shown in the Western blot. The following primary antibodies were used: LC3B (Abcam Cat# ab192890, RRID:AB_2827794), p62 (Abcam Cat# ab109012, RRID:AB_2810880), ULK1 (Cell Signaling Technology Cat# 8054 (RRID:AB_11178668), p-ULK1(Cell Signaling Technology Cat# 5869, RRID:AB_10707365), Beclin-1 (Cell Signaling Technology Cat# 3495, RRID:AB_1903911), LAMP2 (Abcam Cat# ab125068, RRID:AB_10971511), β-actin (Transgen Biotech Cat# HC201, RRID:AB_2860007), and GAPDH (Transgen Biotech Cat# HC301, RRID:AB_2629434).

Electron microscope analysis

After intermittent starvation and SMF treatment, Min6 cells were collected and fixed in 3% glutaraldehyde (containing 2% paraformaldehyde) at room temperature for 1 h, followed by overnight incubation at 4 °C and subsequent washing with PBS. The cells were then fixed twice in PBS containing 1% osmium tetroxide and 1.5% potassium ferrocyanide at room temperature and exposed to 2% uranyl acetate solution. After PBS rinsing, the cells underwent graded ethanol dehydration and were embedded in Spurr low viscosity media. The samples were polymerized overnight at 70 °C. Using Leica Ultra CUT UC7, 100 nm ultrathin sections were cut onto copper grids. After staining with 0.2% lead citrate, the

ultrathin sections were observed using a 120 KV electron microscope (Tecnai G2 SPIRIT BioTWIN, FEI Corporation, RRID:SCR_021365).

Actin proteins (rabbit skeletal muscle actin, Cytoskeleton, Cat# AKL99) was diluted to 3.0 μM in G-buffer (5 mM Tris-HCl [pH 8.0], 0.2 mM $CaCl_2$, 0.2 mM ATP, and 0.5 mM dithiothreitol). The solution was then placed on ice for 1 h and centrifuged at 14,000 rpm, 4 °C for 20 min to remove residual actin oligomers. Next, 10× polymerization buffer (500 mM KCl, 20 mM $MgCl_2$, and 10 mM ATP) was added to induce actin polymerization. The samples were then subjected to either Sham or SMF treatment for 5 min at room temperature under GMF conditions before TEM measurement. For transmission electron microscopy (TEM) experiments, the samples were added to a 200-mesh grid (20 s for ion sputtering) and incubated for 90 s. After grid drying, 1% uranyl acetate was added and incubated for 90 s. The grid was then stained three times with dye, air-dried, and imaged by a electron microscope operated at 200 kV (Talos F200X, FEI Corporation, RRID:SCR_019907).

Open field test (OFT)

The open-field test apparatus (SA215, SANS, China, RRID:SCR_015938) consists of a white plastic board (1000 × 1000 × 400 mm), divided into four enclosed square spaces, allowing simultaneous testing of 4 mice at a time. The enclosed open field is partitioned into a central zone and a peripheral zone using 25 beams of infrared arrays, with the central area defined by the central 9 squares. At the beginning of the experiment, the mice are gently placed in the center of the square space and allowed to freely explore the apparatus for 5 minutes. The entire activity is recorded by a camera installed at the top of the device

and connected to a computer, with synchronized data collected and analyzed using the ANY-Maze video tracking system (Stoelting, USA, RRID:SCR_014289) installed on the computer. After each trial, the open-field apparatus is wiped clean with 75% ethanol.

Mechanical withdrawal threshold (MWT) test

The mechanical withdrawal threshold of db/db mice was measured using a Von Frey aesthesiometer (IITC, USA, RRID:SCR_021751). Each mouse was placed individually in a testing chamber and allowed to adapt to the environment freely for 20 minutes. The Von Frey aesthesiometer was used to stimulate the left hind paw of the mouse. Each mouse underwent 5 repetitions of the stimulus, and the force required for the mouse to rapidly withdraw its paw in response to the stimulus was recorded. At least 5 minutes of rest was given between each stimulation.

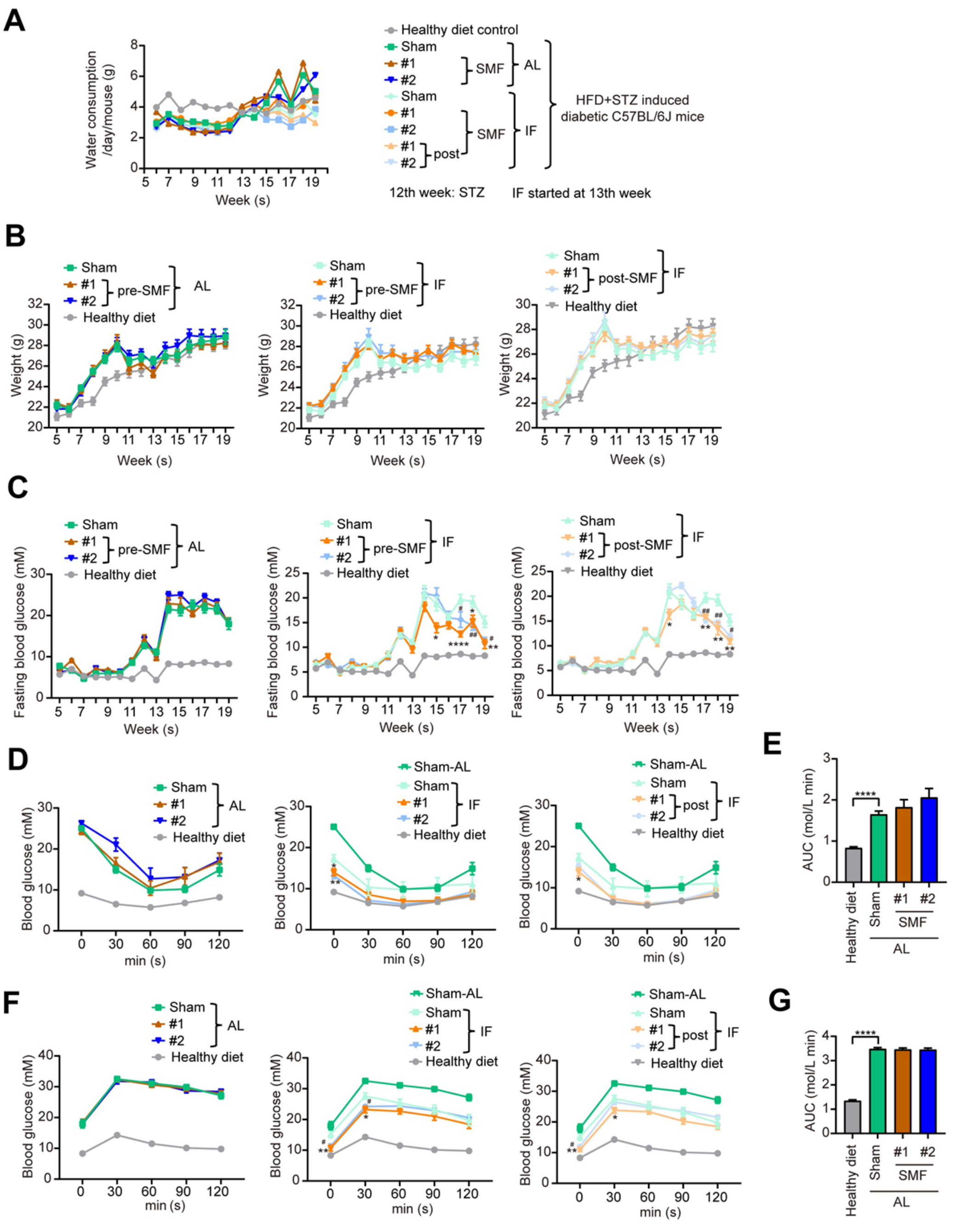

Fig. S1. SMF+IF ameliorates diabetic symptoms in HFD+STZ-induced C57BL/6J mice. (A) Mice water consumption (*n* = 8-10 mice/group). **(B)** Mice weight change (n = 8-10 mice/group). **(C)** Fasting blood glucose curve (*n* = 8-10 mice/group). **(D)** Intraperitoneal insulin tolerance test (IPITT) (*n* = 8-9 mice/group). **(E)** Glucose area under the curve (AUC) of IPITT (*n* = 8-9 mice/group). **(F)** Intraperitoneal glucose tolerance test (IPGTT) (*n* = 8-9 mice/group). **(G)** AUC of IPGTT (*n* = 8-9 mice/group). All data are presented as mean ± SEM and analyzed by GraphPad Prism 9.0. * represents the significance between IF sham control and SMF #1+IF, # represents the significance between IF sham control

and SMF #2+IF. *$P < 0.05$, **$P < 0.01$, ****$P < 0.0001$ and #$P < 0.05$, ##$P < 0.01$, two-tailed Student's *t*-test (C-G).

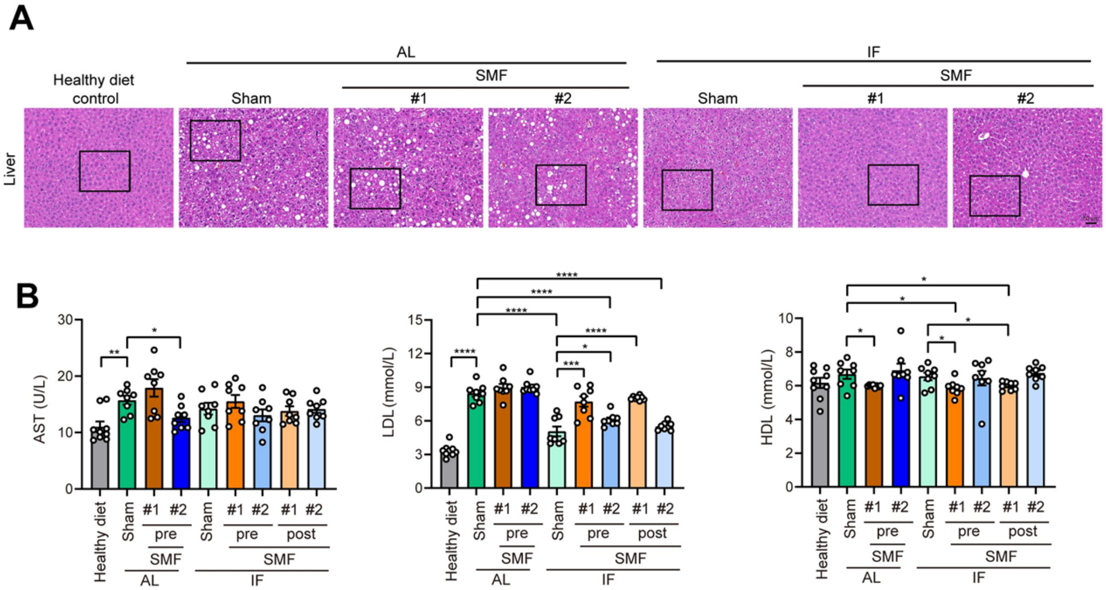

Fig. S2. SMF+IF ameliorates HFD+STZ-induced abnormal hepatic functions of C57BL/6J mice. (A) Representative HE images of mice liver (The box area is partially enlarged as shown in Figure 2G, $n = 3$ mice/group). **(B)** Serum AST, LDL and HDL were measured ($n = 8\text{-}9$ mice/group). All data are presented as mean ± SEM and analyzed by GraphPad Prism 9.0. *$P < 0.05$, **$P < 0.01$, ***$P < 0.001$, and ****$P < 0.0001$, two-tailed Student's *t*-test (B). AST, aspartate transaminase; LDL, low density lipoprotein; HDL, high density lipoprotein.

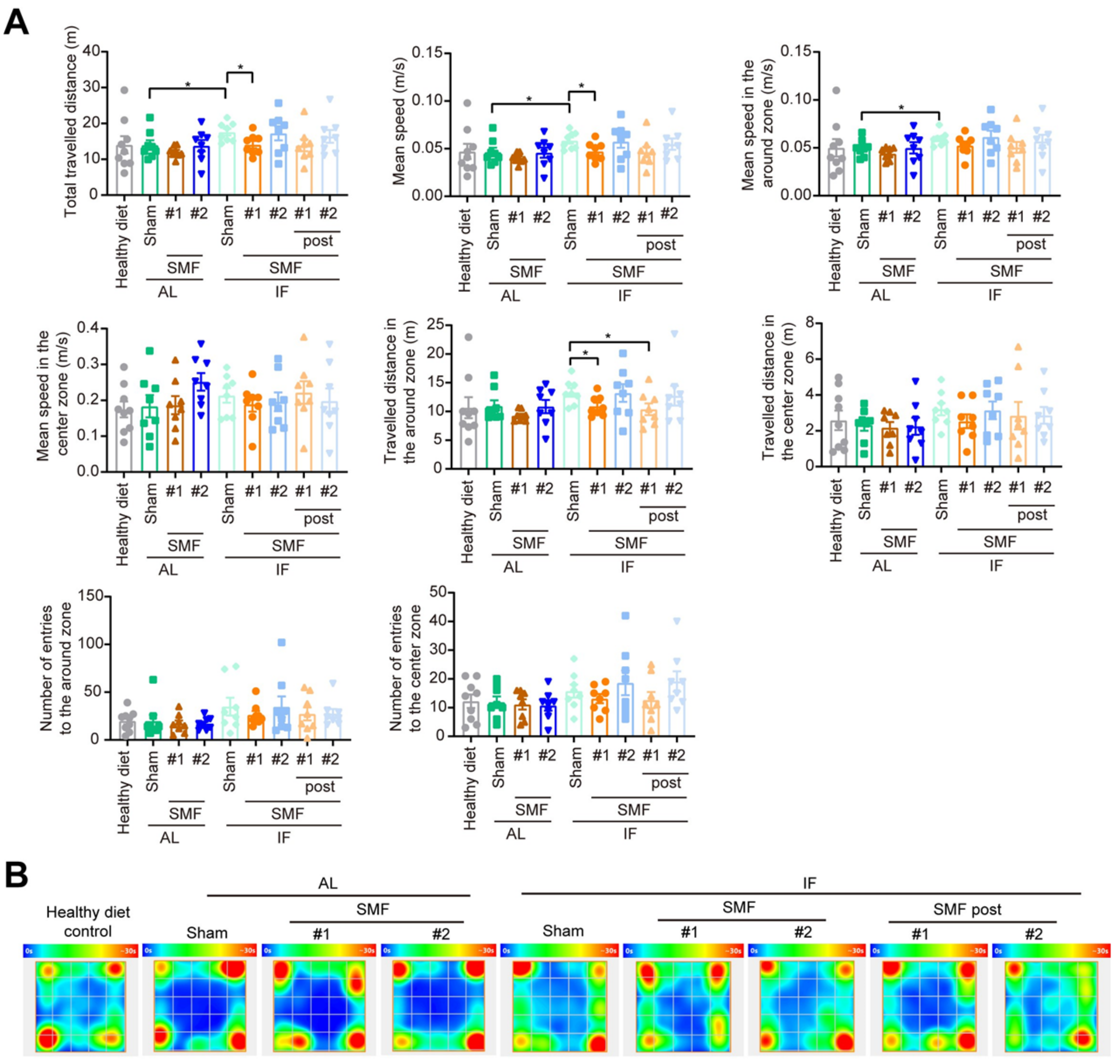

Fig. S3. SMF+IF have a trend towards promoting exploratory behavior and activity abilities in HFD+STZ induced C57BL/6J diabetic mice. (A) The open field test (OFT) related indicators (*n* = 8-9 mice/group). **(B)** The OFT heatmap (*n* = 8-9 mice/group). All data are presented as mean ± SEM and analyzed by GraphPad Prism 9.0. **P* < 0.05, two-tailed Student's *t*-test (A).

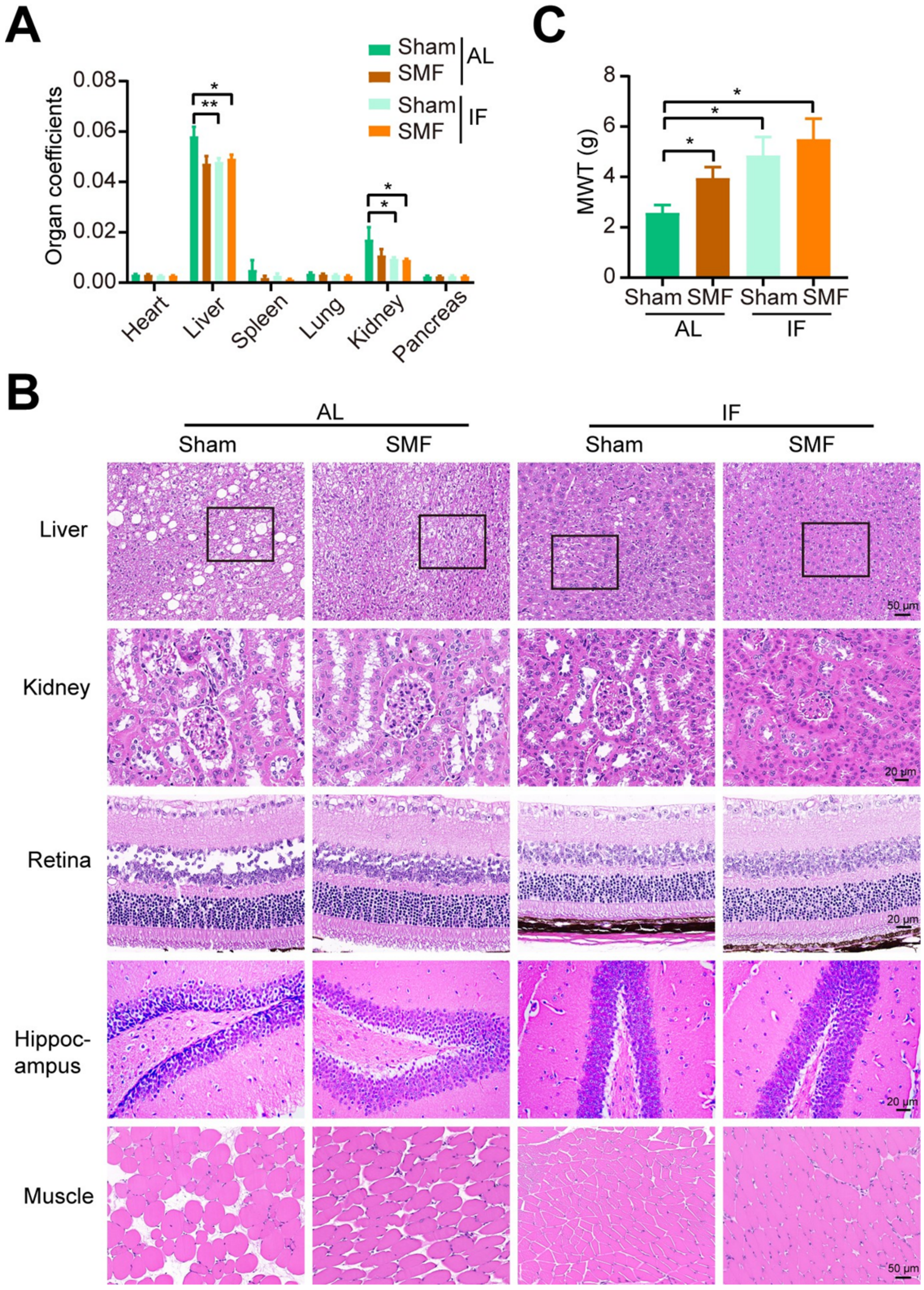

Fig. S4. Static magnetic field combined with intermittent fasting reduces hepatic lipid accumulation and multiple organ lesions in db/db mice. (A) Organ coefficient of db/db mice (It represents the ratio of the organ weight to the weight of the whole body, *n* = 3-9 mice/group). **(B)** Representative HE images of mice liver, kidney, retina, hippocampus and muscle (The box area is partially enlarged as shown in Figure 4D, *n* = 3 mice/group). **(C)** Mechanical withdrawal threshold (MWT) of db/db mice (*n* = 6-9 mice/group). All data are

presented as mean ± SEM and analyzed by GraphPad Prism 9.0. *$P < 0.05$, **$P < 0.01$, two-tailed Student's *t*-test (A, C).

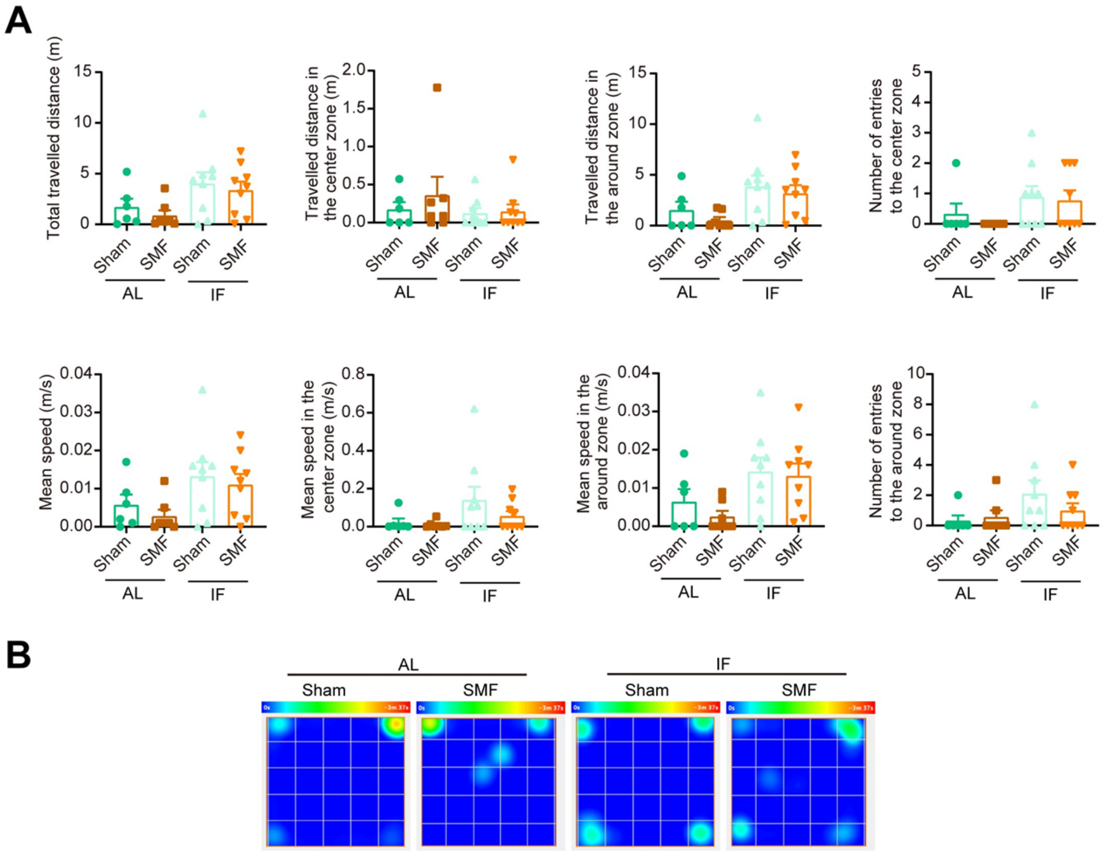

Fig. S5. SMF+IF have a tendency to improve the exercise and exploration ability in db/db diabetic mice. (A) The open field test (OFT) related indicators (*n* = 6-9 mice/group). **(B)** The OFT heatmap (*n* = 6-9 mice/group). All data are presented as mean ± SEM and analyzed by GraphPad Prism 9.0.

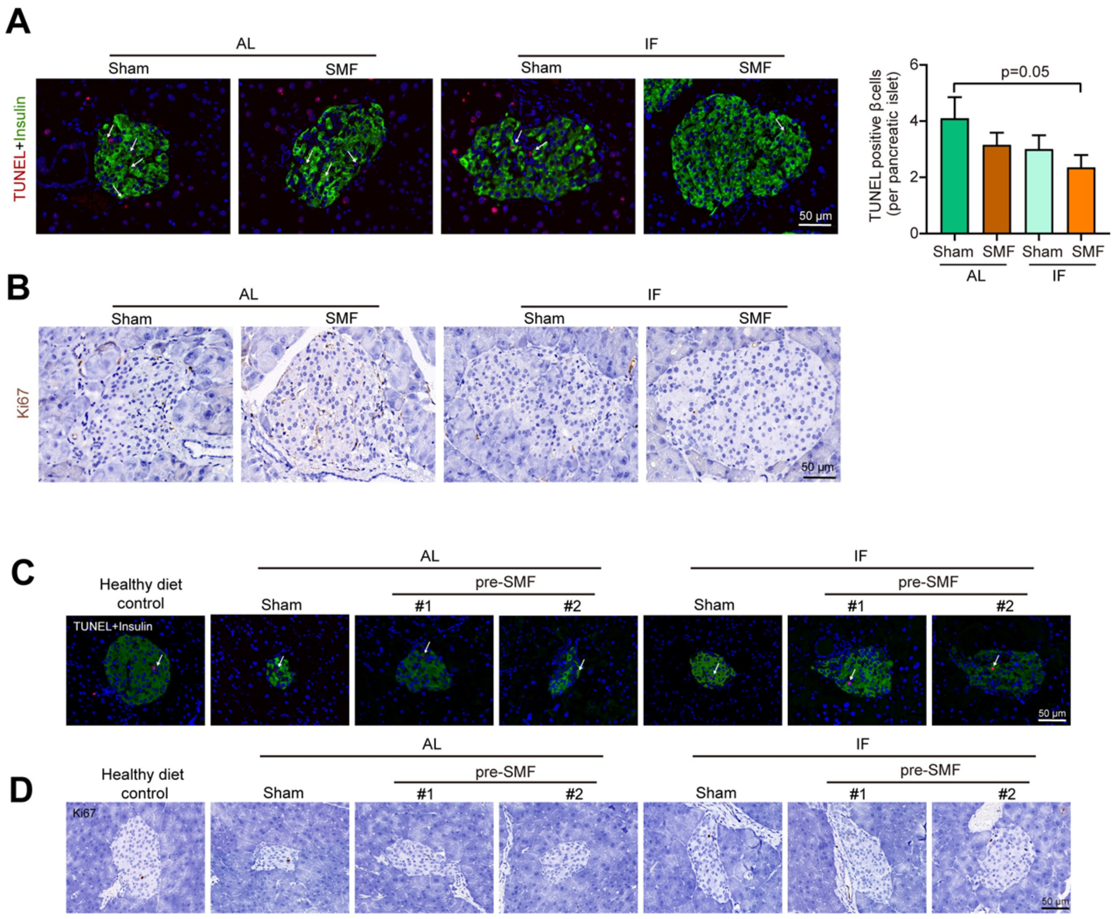

Fig. S6. Apoptosis and proliferation in pancreatic islets of db/db mice and HFD+STZ-induced C57BL/6J diabetic mice. **(A)** Representative TUNEL (red, see arrows) and beta cell (green, anti-insulin) immunofluorescence images and quantifications of db/db mice pancreatic islets (*n* = 20 islets/group, 3 mice/group). **(B)** Representative Ki67 immunohistochemical images of db/db mice pancreatic islets (*n* = 3 mice/group). **(C)** Representative TUNEL (red, see arrows) and beta cell (green, anti-insulin) immunofluorescence images of C57BL/6J mice pancreatic islets (*n* = 3 mice/group). **(D)** Representative Ki67 immunohistochemical images of C57BL/6J mice pancreatic islets (*n* = 3 mice/group). All data are presented as mean ± SEM and analyzed by GraphPad Prism 9.0. $P = 0.05$, two-tailed Student's *t*-test (A).

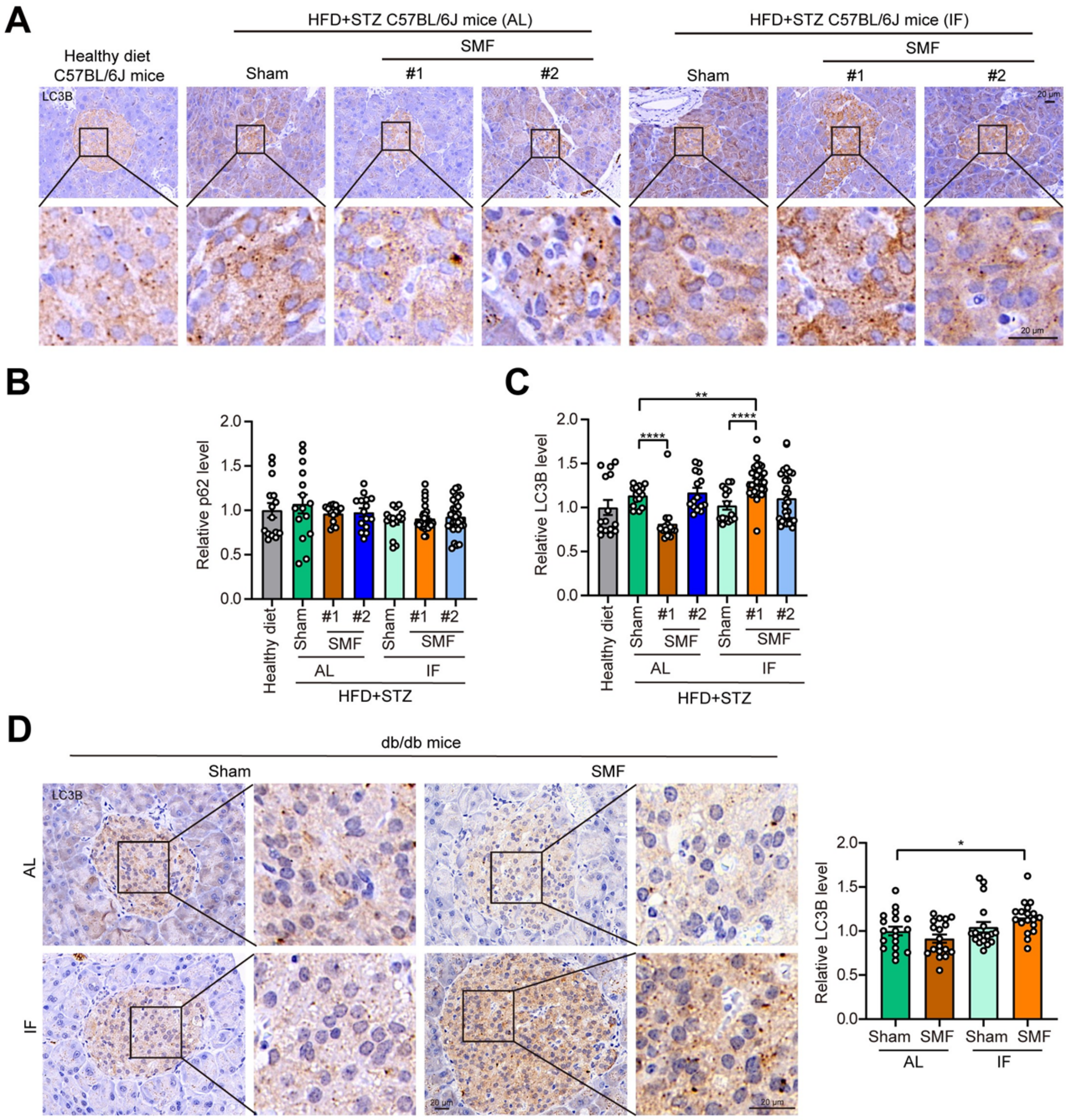

Fig. S7. LC3B staining of HFD+STZ-induced C57BL/6J mice and db/db mice pancreatic islets. (A) Representative LC3B immunohistochemical images of pancreatic islets from HFD+STZ-induced C57BL/6J mice (*n* = 3 mice/group). **(B)** Quantification of p62 level of pancreatic islets from HFD+STZ-induced C57BL/6J mice (*n* = 15-30 islets/group, 3 mice/group). **(C)** Quantification of LC3B level of pancreatic islets from HFD+STZ-induced C57BL/6J mice (*n* = 15-30 islets/group, 3 mice/group). **(D)** Representative LC3B immunohistochemical images and quantification of pancreatic islets from db/db mice (*n* = 18 islets/group, 3 mice/group). All data are presented as mean ± SEM and analyzed by GraphPad Prism 9.0. ***P* < 0.01, and *****P* < 0.0001, two-tailed Student's *t*-test (C, D).

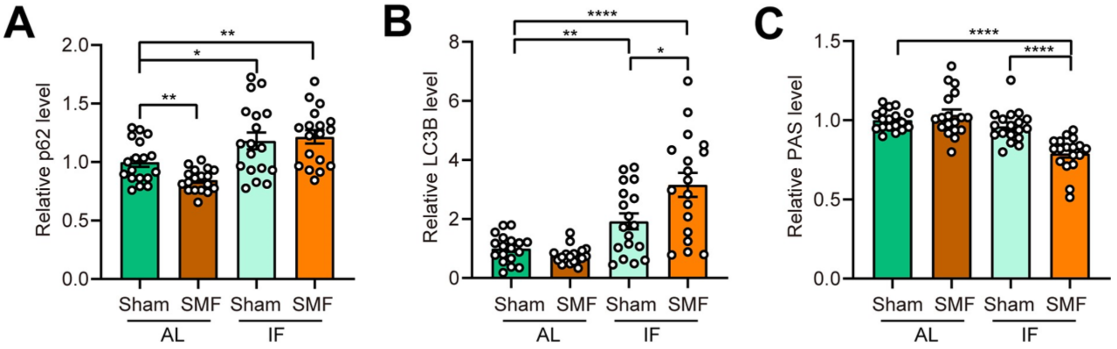

Fig. S8. The quantifications of immunohistochemical and PAS staining of db/db mice liver. (A) Quantification of p62 level of liver from db/db mice (*n* = 3 mice/group). **(B)** Quantification of LC3B level of liver from db/db mice (*n* = 3 mice/group). **(C)** Quantification of PAS level of liver from db/db mice (*n* = 3 mice/group). All data are presented as mean ± SEM and analyzed by GraphPad Prism 9.0. **P* < 0.05, ***P* < 0.01, and *****P* < 0.0001, two-tailed Student's *t*-test (A-C).

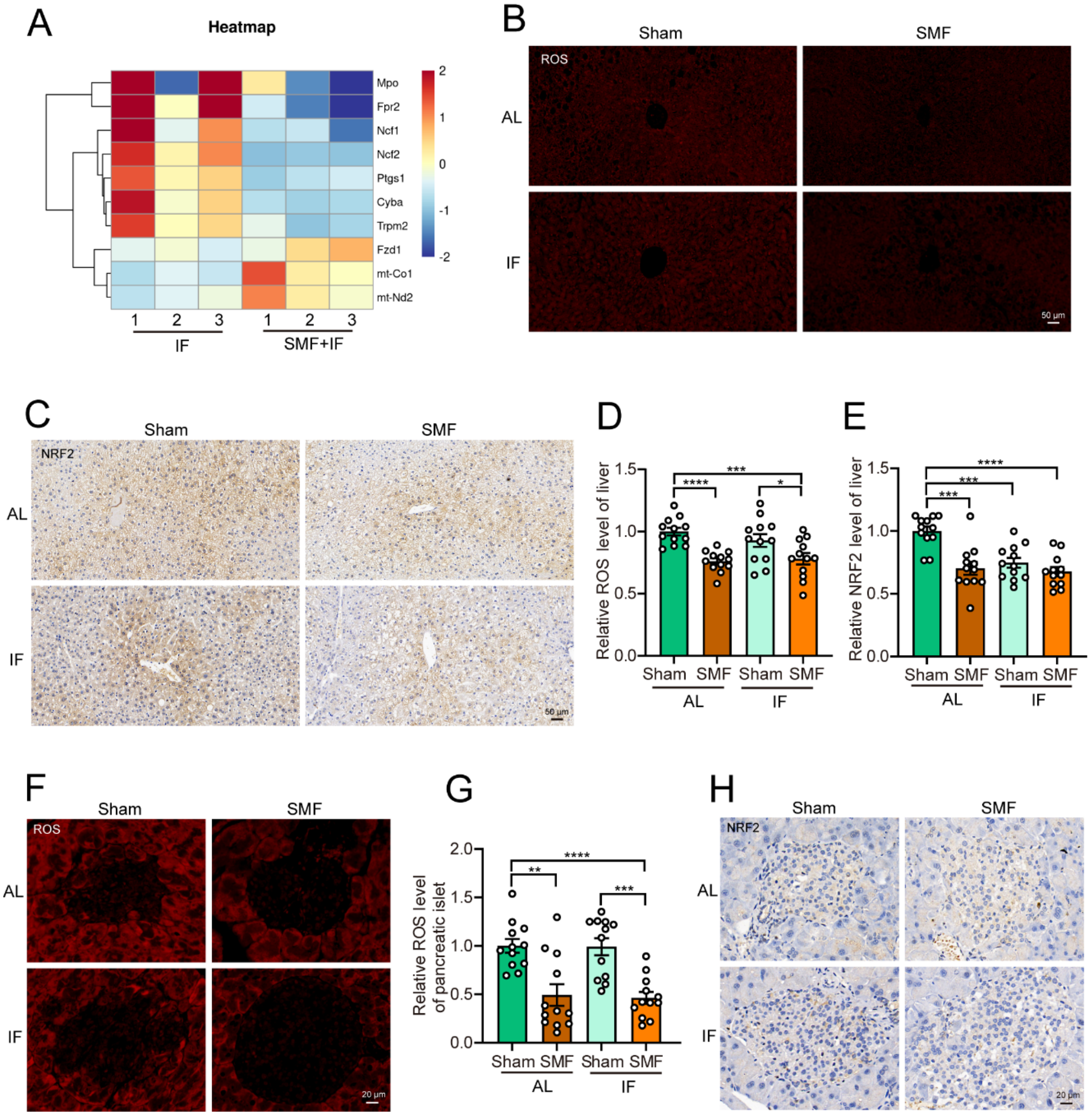

Fig. S9. SMF alleviates oxidative stress in the islets and liver of db/db mice. (A) Heat map of the differential expression genes from SMF+IF vs. IF mice. **(B)** Representative DHE fluorescence images of liver from db/db mice (n = 3 mice/group). **(C)** Representative NRF2 immunohistochemical images of liver from db/db mice (n = 3 mice/group). **(D)** Quantification of ROS level of liver from db/db mice (n = 3 mice/group). **(E)** Quantification of NRF2 level of liver from db/db mice (n = 3 mice/group). **(F)** Representative DHE fluorescence images of pancreatic islets from db/db mice (n = 3 mice/group). **(G)** Quantification of ROS level of pancreatic islets from db/db mice (n = 12 islets/group, 3 mice/group). **(H)** Representative NRF2 immunohistochemical images of pancreatic islets from db/db mice (n = 3 mice/group). All data are presented as mean ± SEM and analyzed by GraphPad Prism 9.0. *$P < 0.05$, **$P < 0.01$, ***$P < 0.001$, and ****$P < 0.0001$, two-tailed Student's t-test (D-E, G).

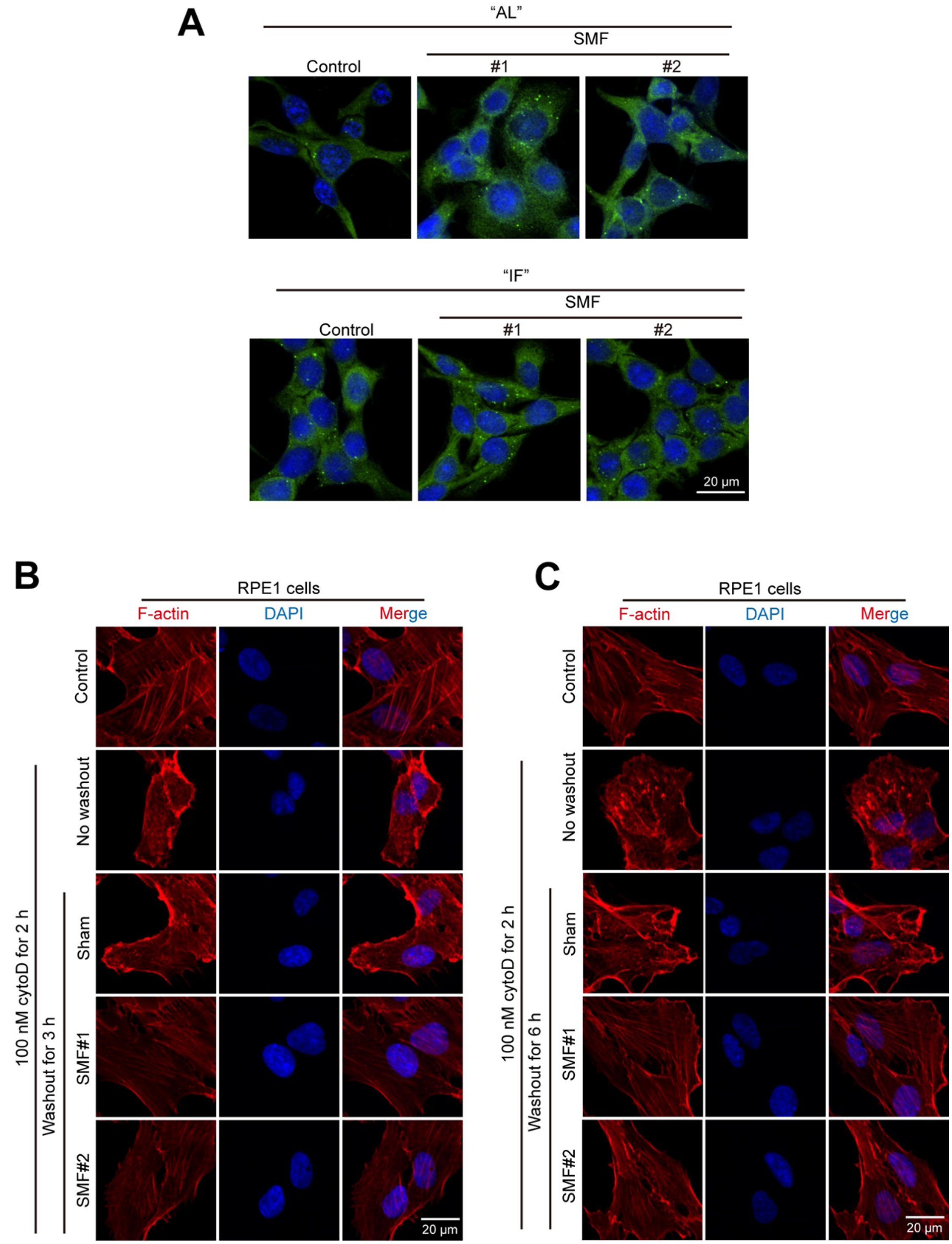

Fig. S10. LC3B immunofluorescence staining of Min6 cells and actin immunofluorescence staining of RPE1 cells. (A) LC3B immunofluorescence staining of min6 cells. Intermittent starvation of Min6 cells using 1% serum starvation was carried out for 48 h with simultaneous SMF treatment for 36 h. (Scale bar: 20 μm). **(B)** RPE1 cells were treated with 100 nM cytoD for 2 h, with or without additional washout to allow recovery for 3 h with Sham or SMF, before they were fixed and stained with phalloidin

and DAPI (Scale bar, 20 μm). **(C)** RPE1 cells were treated with 100 nM cytoD for 2 h, with or without additional washout to allow recovery for 6 h with Sham or SMF, before they were fixed and stained with phalloidin and DAPI (Scale bar, 20 μm).

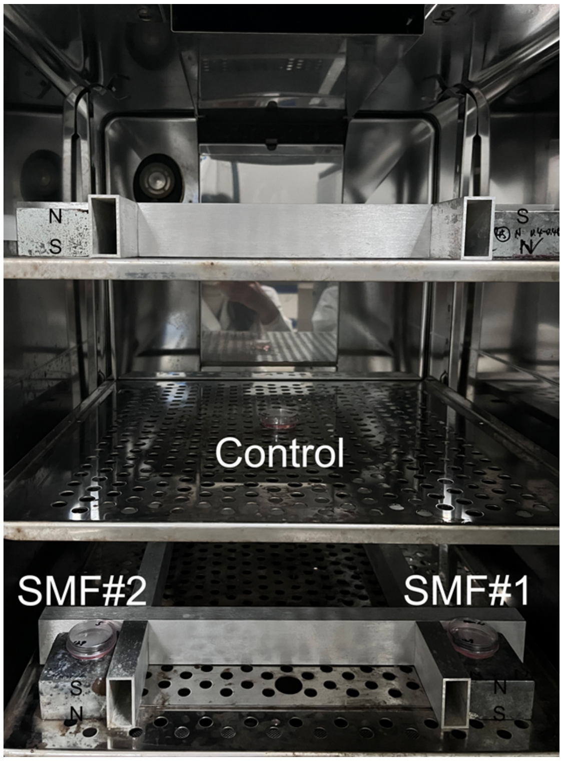

Fig. S11. Cell experimental setup. The cell culture dishes were placed above the north (N) or south (S) pole of the magnets, which provide SMF#1 and SMF#2. The control group was placed inside the same cell culture incubator but far away from the magnets. The measured magnetic flux density of control group is ~0.0001 T, which is ~5000 times lower than the SMF group using a 0.5 T SMF.

Class description	Ingredients	Grams
Protein	Casein, Lactic, 30 Mesh	200.00 g
Protein	Cystine, L	3.00 g
Carbohydrate	Lodex 10	125.00 g
Carbohydrate	Sucrose, Fine Granulated	72.80 g
Fiber	Solka Floc, FCC200	50.00 g
Fat	Lard	245.00 g
Fat	Soybean Oil, USP	25.00 g
Mineral	S10026B	50.00 g
Vitamin	Choline Bitartrate	2.00 g
Vitamin	V10001C	1.00 g
Dye	Dye, Blue FD&C #1, Alum. Lake 35-42%	0.05 g
	Total:	773.85 g

Table S1. The formulation of HFD.
